# Epileptogenesis and Epilepsy Treatment: Advances in Mechanistic Understanding, Therapeutic Approaches, and Future Perspectives

**DOI:** 10.3390/ijms27031175

**Published:** 2026-01-23

**Authors:** Akbota Mazhit, Burkitkan Akbay, Alexander Trofimov, Orynbassar Karapina, Serick Duysenbi, Tursonjan Tokay

**Affiliations:** 1 Department of Medicine, Nazarbayev University School of Medicine, Kerey-Zhanibek khandar 5/1, Astana 010000, Kazakhstan; 2 Biology Department, School of Sciences and Humanities, Nazarbayev University, Kabanbay Batyr 53, Astana 010000, Kazakhstan; 3 Neurosurgery Department, National Scientific Medical Center, Abylaikhan 42, Astana 010000, Kazakhstan

**Keywords:** drug-resistant epilepsy, mechanisms of epilepsy, biomarkers of epilepsy, antiseizure drugs (ASDs), epilepsy surgery, precision medicine, emerging therapy

## Abstract

Epilepsy remains an active and important area of research due to its complex etiology, significant global burden, and variable response to treatment. Current knowledge has provided valuable insights into the underlying molecular mechanisms of the disease and continues to guide the development of novel therapeutic strategies. This review presents a comprehensive overview of the etiologies of epilepsy, as well as traditional and modern medical and surgical treatment approaches, while highlighting future research directions. Peer-reviewed articles retrieved from PubMed and Google Scholar were analyzed and synthesized to produce this review. The etiological complexity of epilepsy arises from genetic, metabolic, structural, and inflammatory mechanisms, which often coexist rather than act independently. A wide range of anti-seizure drugs (ASDs) is currently available, with many new agents targeting novel mechanisms under development. Surgical approaches, including resection, disconnection, corpus callosotomy, and neuromodulation, are widely used for patients with drug-resistant epilepsy and result in variable seizure outcomes. In addition, minimally invasive techniques such as laser interstitial thermal therapy (LITT), stereoelectroencephalography-guided radiofrequency thermocoagulation, gamma knife radiosurgery, and high-intensity focused ultrasound have gained clinical relevance and continue to be explored. Emerging technologies, including artificial intelligence, machine learning, and precision medicine, offer promising directions for future research. Although several potential biomarkers have been identified, none are yet established for routine clinical use. Continued investigation is essential to improve understanding of epileptogenesis and to develop safer, more effective therapies.

## 1. Introduction

Epilepsy is an etiologically heterogeneous neurological disorder defined by the predisposition to recurrent, unprovoked seizures, arising from a complex interplay of structural, genetic, metabolic, infectious, inflammatory, and unknown mechanisms [1,2]. Its clinical manifestations, underlying pathophysiology, and response to treatment vary widely across individuals, reflecting the multifactorial and dynamic nature of epileptogenesis [1].

Anti-seizure drugs (ASDs) have been the cornerstone treatment of epileptic seizures, and there are currently over 25 ASDs available on the market [3]. However, 20 to 40% of patients develop drug-resistant epilepsy (DRE) and require neuro-surgical intervention [4,5]. Epilepsy surgery has seen substantial advances, with current surgical methods ranging from traditional resection to cutting-edge neuromodulation therapies [6,7] such as novel minimally invasive and non-invasive surgical techniques which are based on radiosurgery, thermal therapy, and high-frequency ultrasound, all of which have been in development [7]. Moreover, innovation in pharmacological treatment of epilepsy has taken a leap, as preclinical and clinical trials test potential drugs targeting new molecular mechanisms, with a focus on repurposing existing drugs or investigating benefits of herbal medicine. In addition to the above-mentioned approaches, the application of novel therapies based on artificial intelligence (AI) and machine learning (ML), the development of precision medicine focusing on gene therapies and individual diet, and biomarker-guided therapies are also promising future directions for the treatment and diagnosis of epilepsy. The current review article provides a comprehensive overview on existing knowledge, recent advances, and future prospects in epilepsy research.

**Figure 1:**
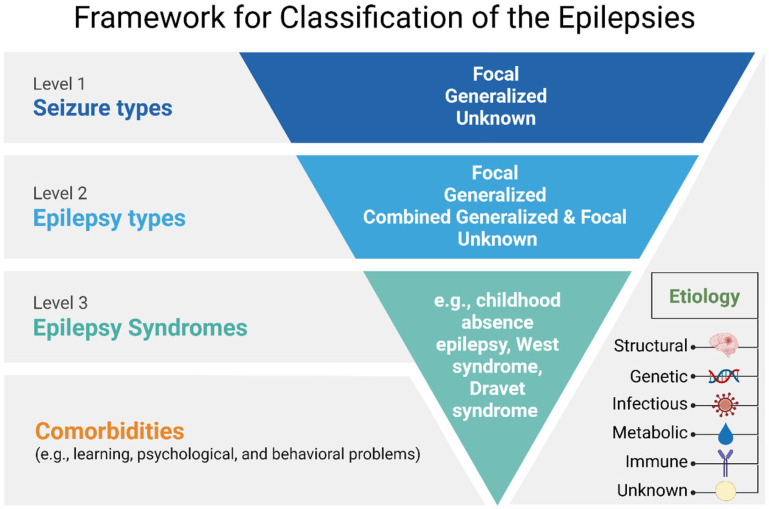
Framework for classification of the epilepsies. This diagram illustrates the ILAE classification system, beginning with seizure type and progressing through epilepsy type and syndrome. Etiology (genetic, structural, metabolic, infectious, immune, or unknown) and comorbidities are included as essential but independent factors. The framework supports a comprehensive, multi-level diagnostic approach to epilepsy.

Epilepsy classification is a key clinical tool for the evaluation of patients with epileptic seizures and their future treatment. The International League Against Epilepsy (ILAE) has recently assembled a number of new definitions and classifications [8]. To facilitate further discussion in this regard, we outlined the updated the classification of epilepsy which broadly includes four categories: (1) seizure types (focal, generalized, or unknown), (2) epilepsy types (focal, generalized, combined focal and generalized, or unknown), resulting epilepsy syndromes, (3) comorbidities, and (4) etiology (structural, genetic, infectious, metabolic, immune, or unknown). (Figure 1).

## 2. Underlying Mechanisms of Epilepsy

Epilepsy is characterized by recurrent seizures caused by abnormally excessive and synchronized neuronal activity in the brain. These disruptions can stem from a variety of causes and underlying mechanisms, including genetic factors, brain metabolism, structural brain abnormalities, acquired brain injuries, inflammations, or even unknown origins. Identifying epileptogenic mechanisms and biomarkers is crucial for developing targeted therapies and improving the lives of individuals with epilepsy. To explore in more depth, the following section will explore the various factors that play a key role in the development of epilepsy.

### 2.1. Genetic Mechanisms

**Figure 2:**
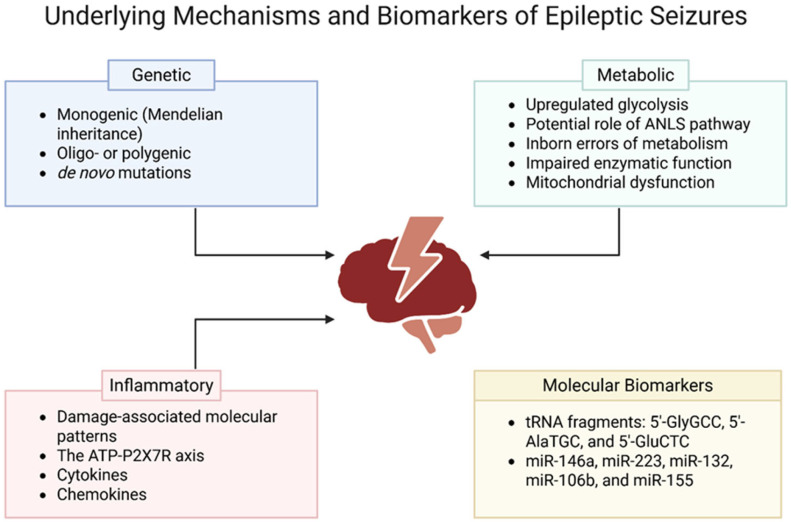
Underlying mechanisms and biomarkers of epileptic seizures. This figure illustrates the main mechanisms contributing to epileptogenesis—genetic, metabolic, and inflammatory—depicted as direct inputs to seizure activity. Molecular biomarkers are shown separately to highlight their role as indicators of epileptogenic processes rather than direct causes. ANLS stands for astrocyte–neuron lactate shuttle.

Genetic mechanisms underlying epilepsy are complex. Our present knowledge indicates that epilepsy comprises a large amount of rare Mendelian subtypes, while the more common forms are mostly oligo- or polygenic, influenced by both common and rare genetic variants [9] (Figure 2). Three primary types of genetic alterations contributing to epileptic disorders have been identified including single gene variants, copy number variants, and common variants [10]. Single gene variants can be inherited in autosomal dominant (AD), autosomal recessive (AR), or X-linked modes of inheritance, which adhere to Mendelian laws [10]. In addition, it has been found that many cases of epilepsy arise from de novo genetic mutations not inherited from either parent [11] (Figure 2). The trio analysis, which involves sequencing of the child and both parents to discover de novo variants, has led to the identification of many epilepsy-related genes [11]. The considerable number of genes identified indicates significant genetic heterogeneity in epilepsy. While 75% of epilepsy cases remain idiopathic, around 25% of epilepsy cases result from an acquired cause such as traumatic brain injury (TBI), infections, brain tumors, or degenerative conditions; even so, phenotypic expressions of acquired epilepsy vary as a result of genetic factors [9].

#### 2.1.1. Generalized Epilepsy

Generalized epilepsy, including genetic generalized epilepsy (GGE), accounts for approximately 15–20% of all epilepsy cases and is characterized by absence, myoclonic, and generalized tonic–clonic seizures [9,12].

However, rare monogenic cases of genetic generalized epilepsy (GGE) have been documented, exhibiting a Mendelian autosomal dominant inheritance pattern. These cases are attributed to pathogenic missense variants of gamma-aminobutyric acid type A receptor subunit alpha1 (*GABRA1*) and gamma2 (*GABRG2*) genes, which encode the α1 and γ2 subunits of the γ-aminobutyric acid type A (GABAA) receptor, respectively [9]. The epilepsy genome-wide association study (GWAS), held by the International League Against Epilepsy (ILAE) and involving 15,212 patients with epilepsy and 29,677 controls, was published in 2018 and identified 16 genome-wide significant loci, 11 of which were attributed to GGE [13]. The study provides novel insights into monogenic epilepsy genes and potential targets for anti-epileptic drugs. Another more recent GWAS meta-analysis was published in 2021, involving 29,944 cases and 52,538 controls with doubling the sample size of cases [14]. The study revealed 26 genome-wide significant loci, 19 of which were attributed to GGE. In addition, behind these 26 loci, 29 genes were identified, of which 17 were associated with epilepsy for the first time. Moreover, the locus 2p16.1 showed the strongest association signal for GGE, and interestingly, rare variants of the gene B-cell lymphoma/leukemia 11A (*BCL11A*) behind this locus were previously associated with epileptic encephalopathy. Notably, among the 26 identified loci are the calcium voltage-gated channel auxiliary subunit α2δ2 (*CACNA2D2*) and the sodium voltage-gated channel α subunit 8 (*SCN8A*), both of which are implicated in epileptic encephalopathy and targeted by anti-epileptic drugs. In addition, the ryanodine receptor 2 gene (*RYR2*) and the cholinergic receptor muscarinic 3 gene (*CHRM3*) have recently been linked to epilepsy.

#### 2.1.2. Focal Epilepsy

Focal epilepsy (FE) accounts for 60% of all epilepsies and has previously been regarded as an acquired type, although a strong genetic component contributes to its pathogenesis [15]. Familial focal epilepsy is a rare syndrome that displays Mendelian inheritance and is inherited in an autosomal dominant mode [16], which is evidenced by the involvement of several genes following autosomal dominant inheritance, such as cholinergic receptor nicotinic alpha 4 subunit (*CHRNA4*), potassium voltage-gated channel subfamily Q member 2 (*KCNQ2*) and 3 (*KCNQ3*), sodium voltage-gated channel subunit alpha 2 (*SCN2A*), and leucine-rich glioma-inactivated 1 (*LGI1*) [15]. Additional genes following autosomal dominant inheritance, such as DEP domain containing 5 (*DEPDC5*), nitrogen permease regulator-like 2 (*NPRL2*) and 3 (*NPRL3*), and encoding components of the GAP activity toward rags 1 (GATOR1) complex, which negatively regulates the mammalian target of rapamycin (mTOR) pathway, have also been discovered to be involved in focal epilepsy pathogenesis [15]. Dysregulation of the mTOR pathway is associated with a myriad of neurological disorders, and elucidation of the genetic components of the mTOR pathway helps to gain insight into focal epilepsy-associated brain pathology [15].

A recent study has identified the post-zygotic rescue of meiotic errors as a novel genetic mechanism underlying focal epilepsy, characterized by brain-specific mosaic chromosomal gains [17]. Genomic analysis of resected brain tissue of five pediatric patients identified an extra parentally derived chromosome 1q allele, enriched in astrocytes. Astrocytes harboring 1q gains exhibited distinct gene expression profiles and hyaline inclusions, revealing a novel genetic link between chromosome 1q alterations and astrocytic inclusions in focal epilepsy [17]. Mosaic variants have long been hypothesized to cause malformations of cortical development (MCD), which are characterized by focal brain lesions and drug-refractory epilepsy [18]. Studies investigating resected brain tissue from patients with hemimegalencephaly, a rare unilateral MCD, revealed somatic mosaic activation of the mTOR signaling pathway: an activating mosaic point mutation in *AKT3*, mosaic copy number gains of chromosome 1q (which contains *AKT3*), and mosaic variants in *PIK3CA* and *MTOR* itself [18,19,20]. Across both studies, these mosaic variants were present only in the affected brain tissue and absent from blood, indicating that the mutations arose post-zygotically and are confined to neural tissue [18,19,20]. Mosaic variants leading to hyperactivation of the mTOR pathway also play a central pathogenic role in both focal cortical dysplasia (FCD) and tuberous sclerosis complex (TSC), which are types of MCD strongly associated with treatment-resistant epilepsy [21,22]. These findings strongly support the model that MCDs can result from mosaic hyperactivation of the mTOR pathway during early neurodevelopment. The absence of these mutations in blood highlights the limitations of peripheral genetic testing and the importance of direct analysis of affected brain tissue. In addition, hyperactivation of the mTOR pathway has direct therapeutic relevance and can be targeted by specific inhibitors such as everolimus.

Beyond the mTOR pathway, abnormal activation of the RAS-RAF-MAPK pathway was found to play an important role in epileptic brain mosaicism. A somatic variant of *KRAS* was associated with the development of FCD, while the *BRAF V600E* mutant was linked to epilepsy-associated brain tumors and hippocampal sclerosis [23,24,25,26]. More recently, mosaic loss-of-function variants of *SLC35A2*, which encodes a UDP-galactose transporter, have been detected in resected brain specimens with neuropathological features of FCD type I, mild malformation of cortical development (mMCD), and mMCD with oligodendroglial hyperplasia in epilepsy (MOGHE) [24]. These findings guide strategies and highlight the challenges of implementing genetic testing for brain-limited mosaic variants, while also informing the development of pathway-targeted therapies for patients with refractory epilepsy.

### 2.2. Metabolic Mechanisms

#### 2.2.1. Impaired Glucose Metabolism

The brain’s metabolisms play a significant role in epileptogenesis. Glucose is the primary energy substrate of the brain and in case of glucose deficiency, ketones and lactate can only partially compensate for glucose [27]. Neuronal activation is associated with a marked increase in glycolysis without a proportional rise in oxidative phosphorylation, leading to lactate production even under aerobic conditions [28] (Figure 2). Lactate may be utilized by neighboring cells and activated neurons particularly during strong and synchronous activity such as seizures [28]. Glucose metabolism fluctuates dynamically across seizure states, with increased glycolytic flux during seizures (ictal hypermetabolism) and reduced glucose utilization during interictal periods (interictal hypometabolism) [29]. During seizures (ictal phase), excessive synchronous neuronal firing acutely increases glucose uptake and glycolytic flux to meet immediate energy demands, whereas during interictal periods, reduced neuronal activity and network dysfunction are associated with persistently decreased glucose metabolism in affected brain regions. A shift towards glycolysis in epileptogenesis even under aerobic conditions, known as aerobic glycolysis, mimics the Warburg effect seen in metabolism of cancer cells [30]. Ictal glucose metabolism can lead to the inadvertent formation of biomass such as gliosis, neurogenesis, and axonal sprouting [31]. Notably, the central role of glucose metabolism in epileptogenesis provides a mechanistic basis for metabolic therapies such as the ketogenic diet which reduces glucose availability and promotes ketone body utilization to modulate neuronal excitability and seizure susceptibility [32].

Neuronal function heavily depends on the instant availability of glucose transported into cells via glucose transporter 3 (GLUT 3) [33]. Although neurons can generate required amounts of ATP to meet their high energy demands, they might also access some metabolic fuel from astrocytes for long-term viability and function [34]. Astrocytic endfeet wrap the surfaces of blood capillaries in the brain and serve as a bridge connecting the capillaries and neurons [35]. According to the astrocyte–neuron lactate shuttle (ANLS) theory, astrocytes obtain glucose from capillaries or extracellular fluid and produce lactate via glycolysis; lactate is then transported via monocarboxylate transporters into neurons to be converted to pyruvate by the activity of lactate dehydrogenase enzyme and fed into the mitochondrial tricarboxylic acid cycle of neurons to produce more energy [36,37] (Figure 2). The ANLS theory explains the optimization of energy metabolism during high synaptic activity, as in epilepsy, and inhibition of lactate dehydrogenase enzyme in ANLS pathway has shown anti-epileptic effects [38,39]. However, the theory remains controversial, as neuronal function is thought to primarily rely on aerobic glycolysis rather than astrocyte-derived lactate during abnormally high activity as in epileptic seizures [40].

#### 2.2.2. Inborn Errors of Metabolism

Several inborn errors of metabolism (IEMs) disrupt neuronal energy homeostasis and are strongly associated with epileptogenesis [41] (Figure 2). Glucose transporter 1 deficiency syndrome (GLUT1-DS) is a rare form of IEM caused by decreased function of the glucose transporter type 1 [42]. GLUT1 deficiency is characterized by defects in the GLUT1 transporter on epithelial cells of brain microvasculature. Consequently, glucose transport into the brain tissue is impaired [43]. GLUT1 deficiency is inherited in an autosomal-dominant manner, and mutations in the solute carrier family 2 member 1 gene (*SLC2A*) that encodes GLUT1 in the mammalian blood–brain barrier are considered its diagnostic hallmark [44]. Patients suffering from GLUT1 deficiency present infantile-onset epilepsy [45]. Another rare form of IEM that may lead to epileptogenesis is the defect in creatine synthesis or transport. Creatine is a precursor for creatine phosphate, a high-energy molecule, and it is transported by creatine transporter I (CT1) from the liver to muscle and brain tissues [46]. Two autosomal recessive disorders characterized by defects in enzymes that catalyze creatine synthesis, L-arginine glycine amidinotransferase (AGAT) deficiency, and guanidine acetate methyltransferase (GAMT) deficiency are associated with epilepsy and severe epileptic encephalopathy [46]. Cerebral CT1 deficiency is inherited in an X-linked pattern and caused by mutations in the *SLC6A8* gene encoding the creatine transporter [47]. Studies have shown that CT1 deficiency not only causes intellectual disability but also causes epileptic seizures in male [46]. In addition, pyridoxine-dependent epilepsy (PDE) is another rare form of IEM that is associated with epileptic seizures.

Pyridoxine-dependent epilepsies (PDEs) result from impaired availability of pyridoxal 5′-phosphate (PLP), a cofactor essential for neurotransmitter synthesis, including GABA production. Mutations in *ALDH7A1*, *PNPO*, or *PLPBP* disrupt PLP metabolism or homeostasis, leading to excitatory–inhibitory imbalance and seizures [48]. Finally, other IEMs shown to initiate epileptogenesis are cerebral folate deficiency characterized by defects in folate transporter and low levels of 5-methyltetrahydrofolate in the brain but adequate folate levels in peripheral organs, and nonketotic hyperglycinemia, an inborn error of glycine cleavage, leading to the excessive accumulation of glycine mainly in the central nervous system and the cerebrospinal fluid [49,50].

#### 2.2.3. Mitochondrial Dysfunction

Mitochondrial impairment is a key contributor to epileptogenesis. Reduced activity of several tricarboxylic acid (TCA) cycle enzymes, including pyruvate dehydrogenase and α-ketoglutarate dehydrogenase, has been observed following acute seizures and status epilepticus [51,52,53,54,55] (Figure 2). Deficits in oxidative phosphorylation, particularly complex I dysfunction, are prominent in epilepsy, especially in temporal lobe epilepsy [56].

Seizure-induced oxidative stress further exacerbates mitochondrial dysfunction. Excessive production of reactive oxygen species (ROS), coupled with impaired antioxidant defenses such as superoxide dismutase 2 and glutathione, contributes to progressive mitochondrial damage [34,57]. Production of neurotoxic ROS may inhibit the complex I and subsequently induce ROS production, a pathologic cycle that increases oxidative stress and propagates damage from seizure [58]. Seizure activity may excessively activate N-methyl-D-aspartate (NMDA) receptors, which leads to the disruption of calcium metabolism and toxic accumulation in the brain, further compromising mitochondrial health and activating necrotic or apoptotic pathways [34]. Mitochondrial dysfunction can result in a wide range of neurological disorders including mitochondrial epilepsy, a neuropathological condition characterized by deficiency in mitochondrial complex I and complex IV subunits in neurons and reactive astrocytes [59,60].

Taken together, the aforementioned evidence suggests that understanding the underlying metabolic mechanisms is crucial in management and treatment of epilepsy.

### 2.3. Inflammatory Mechanisms

#### 2.3.1. Damage-Associated Molecular Patterns

Inflammatory mediators play a critical role in seizures and epileptogenesis. Damage-associated molecular patterns (DAMPs) are endogenous molecules released following tissue injury and are recognized by pattern-recognition receptors (PRRs), triggering sterile inflammatory responses [61]. (Figure 2). High-mobility group protein 1 (HMGB1) is a DAMP that is released by damaged, apoptotic, or necrotic cells under pathological conditions [62]. HMGB1 is a pro-inflammatory molecule that acts upon two PRRs, namely receptor for advanced glycation end-products (RAGE) and toll-like receptor 4 (TLR4), both of which activate a number of transcription factors including nuclear factor-kappa B (NF-κB), contributing to the induction of immune cascades [63]. The HMGB1-TLR4 axis plays a particularly prominent role in epileptogenesis, as it can trigger the phosphorylation of N-methyl-D-aspartic acid receptor (NMDAR) by Src-family tyrosine kinase, increasing calcium ion influx in neurons and contributing to seizures and cell loss [64]. HMGB1 has been found to compromise the integrity of the blood–brain barrier (BBB) via either TLR4 or RAGE [65,66]. HMGB1 can contribute to drug-resistant epilepsy by enhancing P-glycoprotein (a multidrug efflux transporter) in the endothelial barrier of the BBB, a process mediated by TLR4 or RAGE activation [67].

The release of HMGB1 into the extracellular environment is strongly associated with ATP. Under pathological conditions such as trauma, ischemia, hypoxia, and seizures, extracellular ATP concentrations rise, activating the purinergic ligand-gated ion channel 7 receptor (P2X7R), which in turn induces NOD-like receptor protein 3 (NLRP3) inflammasome, resulting in the release of HMGB1 [68]. Moreover, ectonucleotidases cleave extracellular ATP, producing adenosine 50-diphosphate and adenosine, which are known to cause epileptic seizures by triggering inflammatory pathways [69]. The ATP-P2X7R axis also contributes to the generation of reactive oxygen species (ROS) in microglia and the release of prostaglandin E2 (PGE2) [70,71]. (Figure 2). ROS are known to mediate seizure-induced neuronal damage and contribute to pro-inflammatory cytokine production through NF-κB and mitogen-activated protein kinase (MAPK) transcription factors during microglial overactivation [72]. Upregulated biosynthesis of PGE2 after seizure may induce P-glycoprotein at the BBB, resulting in poor efficacy of ASDs [73]. PGE2, the major product of activated cyclooxygenase enzyme-2 (COX-2), has been reported to enhance presynaptic glutamate release by binding to G protein-coupled receptors (GPCRs) and increasing calcium ion influx, thereby inducing neuronal hyperexcitation [73]. In addition, expression of cyclooxygenase-1 (COX-1) enzyme was found to be substantially higher in microglia than in either neurons or astrocytes in hippocampal specimens of patients with drug-resistant mesial temporal lobe epilepsy (MTLE) [74].

#### 2.3.2. Cytokines

In physiological states, cytokines exist in the brain at low levels; however, their concentrations can rise after an epileptogenic insult to the brain such as hypoxia, stroke, infection, and injury [75]. Pro-inflammatory cytokines such as interleukin-1 beta (IL-1β), interleukin-6 (IL-6), and tumor necrosis factor alpha (TNF-α) are known to be the major cytokines involved in epileptogenesis [76,77] (Figure 2). Activated microglia, astrocytes, endothelial cells of the BBB, leukocytes extravasated into the brain, as well as neurons can secrete IL-1β during epileptogenesis [78]. Concurrently, the compromise of BBB during epileptic episodes facilitates the infiltration of peripheral immune components, which notably complements proteins. These proteins interact with complement receptors expressed on neurons and glial cells, thereby stimulating the release of pro-inflammatory cytokines and initiating microglia-driven synaptic remodeling [79,80,81]. As part of its pro-inflammatory activity, IL-1β was found to down-regulate the expression of complement factor H (CFH), a key inhibitor of the alternative pathway of complement activation [82]. Reduced expression of CFH leads to excessive complement activation and sustained inflammation, which contributes to epileptogenesis [82]. Upon binding to its receptor, IL-1R, IL-1β induces Src-kinase-mediated phosphorylation of the NR2B subunit of the NMDAR, which increases the calcium ion influx in neurons, leading to hyperexcitation [78]. The IL-1β-IL-1R1 pathway activates MyD88-dependent, ceramide-mediated Src-kinases, affecting the neuronal A-type K+ currents and reducing the synaptic release of GABA, which ultimately leads to seizure generation [78]. IL-1β has been shown to upregulate matrix metalloproteinase-9 (MMP-9) activity, which contributes to the disruption of tight junction proteins within the blood–brain barrier (BBB), ultimately compromising its structural integrity [83]. In addition, IL-1β - similar to reactive oxygen species (ROS) - has been implicated in the activation of upstream regulators of the mTOR signaling pathway during the process of epileptogenesis [71,84]. Notably, activation of the mTOR pathway has been proposed to exert pro-ictogenic effects, at least in part, by impairing BBB function, as demonstrated in experimental rat models [85].

IL-6 may have both neuroprotective and ictogenic effects [86,87]. Activated microglia, astrocytes, endothelial cells of the BBB, and neurons can express both IL-6 and its receptor IL-6R [86]. Administration of IL-6 has been reported to impede the cytosol-membrane translocation of Gamma-Aminobutyric Acid Type A Receptors (GABAARs) in either direction and selectively decrease GABAergic inhibition while preserving glutamatergic excitation in the temporal cortex of the mouse model [88]. Similarly to IL-6, TNF-α also has dual function. It is mainly expressed by microglia, astrocytes, and endothelial cells [89,90]. TNF-α targets two receptors, TNF-α receptor R1 (p55TNFR) and TNF-α receptor R2 (p75TNFR), opposing activities of which contribute to its dual function [91]. High concentrations of TNF-α were reported to activate p55TNFR propelling ictogenesis in AMPA(α-amino-3-hydroxy-5-methylisoxazole-4-propionic acid)-induced excitotoxic mouse hippocampal slices, while low concentrations of TNF-α activate p75TNFR-producing anticonvulsant effects [92]. However, the activated p75TNFR can impair endothelial cell function and BBB integrity [93]. TNF-α stimulates glutamate release by microglial cells, augments the activity of calcium-permeable AMPA and NMDA glutamate receptors, and inhibits astrocytic glutamate uptake while enhancing GABA uptake, triggering excitatory synaptic activity [81].

TGF-β also plays a role in inflammation during epileptogenesis. As the integrity of the BBB is damaged during ictogenic event, TGF-β signaling pathway mediates serum albumin uptake by astrocytes, leading to neuronal hyperexcitation, expression of pro-convulsant inflammatory agents, down-regulation of gap junction proteins, and impairment of potassium ion and glutamate buffering in astrocytes [63]. Another key contributor to the neuro-inflammatory cascade involved in epileptogenesis is platelet-activating factor (PAF), a major pro-inflammatory lipid, which exerts epileptogenic effects by inducing glutamate release and COX-2 expression [94].

#### 2.3.3. Chemokines

Chemokines are pivotal mediators in neuro-inflammatory processes that direct the trafficking of leukocytes toward sites of inflammation [95]. In individuals diagnosed with temporal lobe epilepsy (TLE), transcriptomic analyses using DNA microarrays have revealed a significant upregulation of chemokines including chemokine (C-C motif) ligand 2 (CCL2), 3 (CCL3), and 4 (CCL4) [94]. (Figure 2). The complement-derived anaphylatoxins C3a and C5a have been shown to enhance the expression of cell adhesion molecules (CAMs), a process that aids in the recruitment and subsequent infiltration of leukocytes into the brain tissue [96]. Furthermore, the C1q-C3 axis has been implicated in the mechanisms underlying epileptogenesis, supported by findings of elevated concentrations of C1q and iC3b—a cleavage product of C3b—in the serum of patients with pharmacoresistant epilepsy [97]. Collectively, these findings highlight the complex interplay of inflammatory mediators in the etiopathogenesis of epilepsy and highlight potential molecular targets for therapeutic intervention.

### 2.4. Molecular Biomarkers

Currently, no prognostic biomarkers have been definitively identified that can reliably predict the onset of epileptic seizures. The identification and validation of such biomarkers would be instrumental in advancing the development of anti-epileptogenic therapies aimed at preventing seizure initiation, thereby significantly enhancing the quality of life for individuals with epilepsy.

In patients with drug-resistant focal epilepsy, elevated plasma levels of three transfer RNA fragments—5′-GlyGCC, 5′-AlaTGC, and 5′-GluCTC—were detected prior to seizure onset and shown to localize within hippocampal and cortical regions, suggesting their release into systemic circulation in response to epileptogenic activity [98] (Figure 2). Comparative analyses demonstrated a significant post-seizure decline in their concentrations across most patient samples, supporting their temporal dynamics and potential utility as prognostic biomarkers of epileptogenesis [98].

Considerable attention has been directed toward microRNA-146a (miR-146a), an astrocyte-enriched molecule that functions as both a diagnostic and prognostic biomarker of epileptogenesis [99] (Figure 2). miR-146a modulates pro-inflammatory signaling via negative feedback on the IL-1R1/TLR4 axis, a pathway implicated in seizure development, and clinical studies consistently show its elevated expression in patients with epilepsy across diverse etiologies compared with healthy controls [68,75,99,100,101,102].

Moreover, other microRNAs involved in neuro-inflammatory and neuroprotective pathways such as miR-223, miR-132, miR-106b, and miR-155 have also shown promise as prognostic and diagnostic indicators of epileptogenesis [99,100,102,103] (Figure 2). Collectively, these findings underscore the potential of circulating non-coding RNAs as molecular biomarkers for early detection and monitoring of epilepsy progression.

### 2.5. Functional Connectivity and Cognition

Epilepsy is increasingly conceptualized as a brain network disorder in which seizures and interictal activity disrupt intrinsic connectivity across distributed neural systems. Functional connectivity (FC) refers to temporal correlations between activity in distinct brain regions and is typically assessed using resting-state functional MRI (rs-fMRI) or combined EEG-fMRI approaches. Altered FC has been associated with deficits in memory, language, attention, and executive function in both focal and generalized epilepsies, indicating that network disturbances contribute to cognitive comorbidity beyond focal lesion effect [104]. Studies using rs-fMRI have demonstrated that resting-state networks such as the default mode network (DMN) and limbic connectivity are disrupted in temporal lobe epilepsy (TLE) and other focal epilepsies. For example, individuals with benign mesial TLE showed bilateral hypoconnectivity between hippocampal and amygdala regions and language/memory networks, with connectivity strength correlating with verbal memory performance, suggesting a direct link between FC alterations and cognitive outcomes [105]. In chronic focal epilepsy, broad functional abnormalities within frontal, temporal, and DMN circuits have also been associated with cognitive impairments including impaired memory and executive control, consistent with classical rs-fMRI findings [104].

Large-scale network analyses further show that aberrant connectivity within and between canonical networks—such as fronto-parietal control, salience, and limbic circuits—relates to domain-specific cognitive deficits across patient cohorts, supporting the notion that epilepsy-related network disruption can compromise cognitive processing even in the absence of persistent seizures [106]. Importantly, FC metrics correlate with neuropsychological performance and may serve as biomarkers for cognitive dysfunction beyond structural pathology alone [106].

## 3. Current Standard Treatment Methods

Current treatment strategies for epilepsy rely on anti-seizure medications as the primary treatment modality, with a diverse array of approved drugs available to cater to various seizure types and patient profiles. For patients with drug-resistant epilepsy, surgical interventions offer additional therapeutic options, such as resection of epileptogenic foci and disconnection procedures, including corpus callosotomy. Neuromodulation techniques, such as vagus nerve stimulation, deep brain stimulation, and responsive neurostimulation, have further expanded therapeutic possibilities for patients who are not candidates for resective surgery. Together, these approaches form the current standard of care aimed at achieving seizure freedom and enhancing the quality of life for patients.

### 3.1. Approved Anti-Seizure Drugs

**Figure 3:**
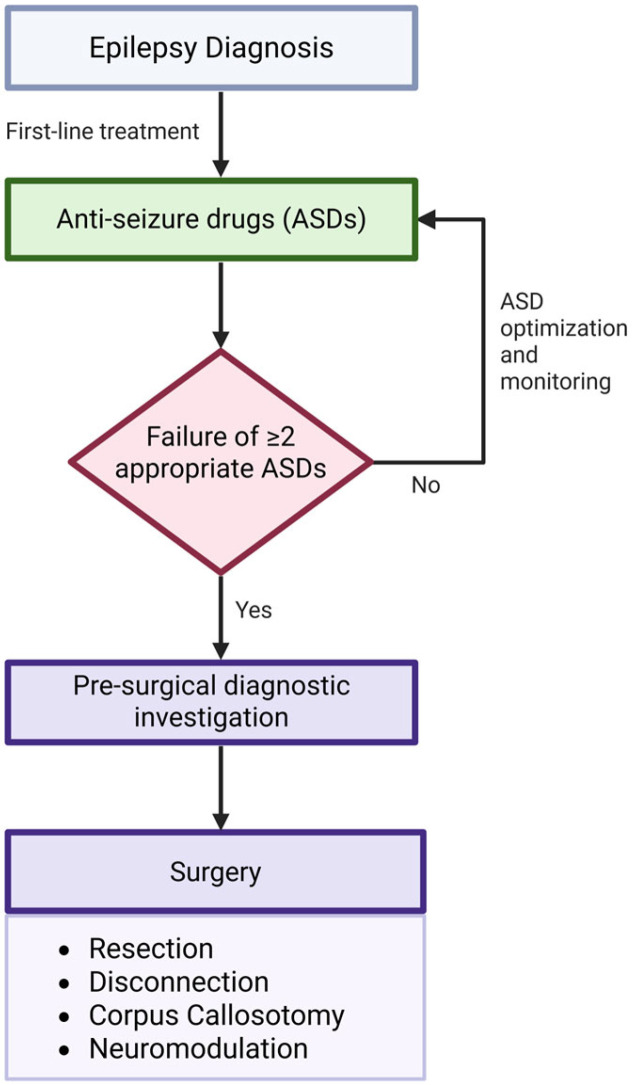
Overview of epilepsy treatment options. Following diagnosis, patients are typically managed with anti-seizure drugs (ASDs) as first-line therapy. If seizures persist despite adequate trials of two or more appropriate ASDs, surgical interventions are considered.

Anti-seizure drugs (ASDs) remain the first-line therapeutic approach for the symptomatic control of epilepsy [107,108]. While commonly associated adverse effects include headache, dizziness, somnolence, fatigue, fever, and nausea, each ASD is characterized by a distinct side effect profile [107]. Older-generation ASDs have been in use for over 40 years, but 30–40% of patients on these monotherapies experience adverse effects that contribute to treatment failure [108]. (Figure 3). Availability and use of ASDs is another concern, as it differs substantially across geographic regions due to regulatory approvals, healthcare infrastructure, cost, and supply dynamics, which contributes to global treatment gaps and highlights the importance of contextualizing ASD discussions within regional access patterns. In many low- and middle-income countries (LMICs), access to newer second- and third-generation ASDs such as lamotrigine, levetiracetam, lacosamide, and perampanel remains limited or sporadic, with treatment dominated by older, more affordable agents such as phenobarbital, carbamazepine, valproate, and phenytoin [109,110,111]. For example, surveys across Asia (including India, Vietnam, Lao PDR, and Myanmar) report that newer ASDs are either unaffordable or entirely unavailable in some settings, whereby essential medicines often stock-out in public facilities and only a limited panel of older ASDs are routinely stocked (e.g., phenobarbital and carbamazepine) [111,112]. Similarly, in sub-Saharan Africa (e.g., Zambia and Madagascar), nearly half of community pharmacies lack even basic ASDs, and treatment gaps remain high due to inconsistent drug supply and affordability barriers, especially in rural areas [113,114]. In contrast, high-income countries such as the United States, and much of Europe, Japan, and Australia, report widespread availability of newer ASDs supported by robust reimbursement schemes, with higher consumption rates of lamotrigine, levetiracetam, and other modern agents [111,115]. These factors drove the development of newer ASDs with novel molecular targets [108]. Over the past three decades, around 20 second- and third-generation ASDs have been approved, offering diverse mechanisms and pharmacokinetics tailored to individual patient needs [108]. (Figure 3). While seizure control is comparable to older ASDs, newer agents generally offer improved tolerability, with fewer neurotoxic side effects, as noted by the American Academy of Neurology (AAN) subcommittee in 2004 [108]. The following table outlines various ASDs along with their proposed mechanisms of action, clinical indications, and specific adverse effect profiles (Supplementary Table S1).

### 3.2. Surgery

Epilepsy surgery has undergone major advancements in recent decades, supported by three randomized controlled trials confirming its effectiveness and acceptable safety profile [116]. Nevertheless, epilepsy surgery continues to be underused in clinical practice [116]. Refractory epilepsy may develop in an estimated one-third of patients who do not respond favorably to two or more ASDs, at which point surgical evaluation should be initiated [117] (Figure 3). Unlike patients with pharmacoresponsive epilepsy, who benefit from the first-line treatment with ASDs, individuals with DRE remain at risk for cumulative physical, cognitive, psychosocial impairment and sudden unexpected death in epilepsy (SUDEP), underscoring the importance of timely surgical referral [117].

Pediatric and adult epilepsy surgery differ markedly because the causes and network behavior of epilepsy differ fundamentally by age. Children typically have genetic, metabolic, or developmental malformations, often producing misleading EEG and rapidly progressive seizures, requiring early, sometimes urgent surgical evaluation and use of invasive monitoring [118]. Adults, by contrast, usually develop epilepsy from acquired focal lesions such as mesial temporal sclerosis, tumors, infections, or post-stroke scars and are considered for surgery only after years of unsuccessful medical therapy, often allowing clearer localization and more focal resections [118]. Surgical risk–benefit profiles also differ young children who have high neuroplasticity, enabling extensive or eloquent-region surgeries, whereas adults face greater risk of postoperative deficits and surgical failure when the epileptogenic zone overlaps the eloquent cortex [118].

Pre-surgical diagnostic investigation begins from assessing ictal semiology, i.e., signs and symptoms of seizure that aid classification of the epileptogenic zone (EZ), and may include non-invasive or invasive techniques [119] (Figure 3). Non-invasive techniques include the following: (1) video-electroencephalogram (EEG) that depicts ictal, interictal, and postictal epileptiform discharges using scalp electrodes; structural magnetic resonance imaging (MRI) that helps reveal cerebral lesions and focal abnormalities; functional magnetic resonance imaging (fMRI) that is particularly useful for pre-surgical differentiation of eloquent cortical areas from potential EZ, preventing postoperative neurological deficits, and investigation of language and motor functional systems; (2) a fludeoxyglucose-18 (FDG) positron emission tomography (PET) scan evaluates interictal impairments and may reveal hypometabolic patterns near suspected EZ in patients with MRI-negative TLE, predicting a favorable surgical outcome; (3) single-photon emission computed tomography (SPECT) scans evaluate ictal brain activity and inform on changes in regional cerebral blood flow (rCBF), a marker of enhanced neuronal activity; (4) pre- and post-surgical neuropsychological and psychiatric investigations are essential and entail the assessment of cognitive functions such as memory, attention, intelligence, language, and pre-existing psychiatric morbidities [120]. In case of failure of non-invasive techniques, invasive diagnostic techniques are employed to precisely identify the EZ: (1) stereo-electro-encephalography (SEEG) makes use of stereotactically implanted intracerebral electrodes at the presumed epileptogenic region, with a low complication rate that is further reduced by robotic assistance; (2) subdural electrodes, though effective for neocortical mapping, require craniotomy and may result in possible complications such as infections, cerebral edema, intracranial hemorrhages, and CSF leakage [120].

Surgical intervention may be contraindicated in certain clinical scenarios, including the presence of genetic generalized epilepsy, progressive neurological or systemic disorders, or unfavorable outcomes from pre-surgical neuropsychological and psychiatric assessments [120].

#### 3.2.1. Resection

Medically refractory epilepsy can be treated with surgical removal of affected brain areas such as mesial temporal lobe resection, including the resection of hippocampus, amygdala, entorhinal cortex, temporal neocortex, or neocortex in the remainder of the brain [121] (Figure 3). While mesial temporal lobe resection remains the most investigated type of epilepsy surgery, there is a paucity of research on the outcomes of extratemporal resection [121,122]. Two randomized controlled trials (RCTs) reported the efficacy of resection in adult patients with pharmacoresistant (resistant to >2 ASDs) epilepsy with clear seizure semiology at the mesial temporal lobe [122]. In one trial, 58% of surgical patients became free of disabling seizures compared with 8% receiving medical therapy, alongside significantly better quality of life (*p* < 0.001) [123]. Surgical complications included a thalamic infarct, infection, and declines in verbal memory [123]. In a second RCT, the outcomes of anteromesial temporal lobe resection were evaluated in patients suffering from intractable mesial temporal lobe epilepsy (MTLE) for no more than two consecutive years [124]. Out of 23 patients in the medical group, none were seizure-free compared to 11 out of 15 seizure-free patients in the surgical group [124]. Furthermore, patients in the surgical group demonstrated significantly higher increases in health-related quality of life than patients in the medical groups at 6, 12, and 18 months, but the improvement in quality of life was not sustained until 24 months [124].

Effects of a range of epilepsy surgeries such as temporal lobectomy, extratemporal lesion resection, hemispherotomy, corpus callosotomy, disconnection, and resection of hypothalamic hamartomas were also evaluated in children and adolescent patients (age ≤ 18 years), showing rather heterogeneous epilepsy etiologies, age of onset, and duration of seizures prior to intervention [122,125]. Evidence from one RCT demonstrated that at 12 months, 77% of patients were seizure-free after surgery compared to 7% of patients who received medical treatment (*p* < 0.001) [125]. Seizure freedom reached 100% for temporal lobectomy and hypothalamic hamartoma resection, 92% for extratemporal resection, and 87% for hemispherectomy [125]. Evidence across trials indicates that more extensive resections generally achieve better seizure control, though the benefit must be balanced against risks of neurological deficits [121].

RCTs comparing extent of mesial temporal resection have produced mixed findings. Total hippocampectomy resulted in higher seizure freedom than partial removal (69% vs. 38%), whereas another trial found that good seizure freedom is associated not with maximal tissue removal but with achieving an adequate resection volume [126,127]. For surgical approaches, one study reported no significant difference between transsylvian and subtemporal surgery (64% vs. 59% seizure freedom) [128].

Despite strong short-term efficacy, long-term seizure freedom after temporal lobe surgery declines to 50–60% at 10 years [122]. Extratemporal outcomes are poorer: a meta-analysis reports seizure freedom in ~45% of frontal and parietal-occipital resections at ≥4–5 years [121,129,130]. Operative mortality is 0.4% for temporal and 1.4% for extratemporal resections [121]. More research is warranted to evaluate the outcome of extratemporal resections.

Recurrence after resection is common. In a cohort of 898 patients, 58% experienced seizure recurrence, attributed to mislocalization of EZ, incomplete resection, or unidentified causes [131]. Incomplete resections can even precipitate postoperative paradoxical deterioration of seizures [131]. Importantly, patients who had multiple reoperations were more likely to have undergone resections on the left hemisphere, which is explained by incomplete resection of the EZ to prevent damage to the eloquent cortex, omitting residual diseased tissue [131]. Right temporal epilepsy allows for more extensive resection, as the risk of postoperative deficits in verbal recall and memory is less significant compared to left-sided resections (20% for right-sided vs. 44% for left-sided) [121]. Although temporal lobe resection may cause cognitive decline, chronic seizure activity may lead to neuropsychological impairments as well [131]. Seizure freedom decreases with each subsequent surgery, and a subgroup of patients may have surgically refractory epilepsy defined by ‘malignant’ epileptogenic network, capable of propagating new EZ at the site of resection [131]. This may be explained by the inherent biological characteristics of the patients (male sex and tendency for secondary generalization), higher frequency of preoperative seizure, EZ localized in the dominant hemisphere, invasive diagnostic techniques, or early seizure recurrence after previous surgery [131]. Careful selection of candidates for reoperations is therefore essential.

#### 3.2.2. Disconnection

Patients who present with multiple epileptic foci (e.g., 60% of pediatric epilepsies) may require multilobar resection, which is frequently accompanied by serious surgical complications such as hematoma, hemianopsia, hydrocephalus, superficial hemosiderosis, shunt-related craniosynostosis, visual field defects, and occasional new motor deficits brought upon by loss of intracranial volume and cavity formation [132,133,134]. Operative disconnection allows functional isolation of the hemisphere or multi-lobe affected by epilepsy, while reducing the risk of complications and preserving vital vascularization of the brain [133] (Figure 3).

Functional hemispherotomy is a type of disconnection surgery prescribed to children with unilateral hemispheric pathologies such as hemimegalencephaly, diffuse hemispheric focal cortical dysplasia, congenital large-vessel stroke, Sturge–Weber syndrome, and Rasmussen’s encephalitis [118,133]. While anatomical hemispherectomy, involving a complete resection of the affected hemisphere, can yield superior seizure control, its higher complication rate favors functional hemispherotomy despite slightly greater risk of early recurrence [133].

Posterior quadrant disconnection (PQD) is a modification of peri-insular hemispherotomy and considered in patients with intractable multilobar epilepsies with seizures arising from temporo-parieto-occipital lobes of the brain [135,136]. Total PQD completely disconnects the temporal, parietal, and occipital lobes, at the same time preserving the sensorimotor cortex, corticospinal fibers, the frontal lobe, and insula, while partial PQD disconnects only particular regions of the posterior quadrant [136]. Both of these procedures yielded a pooled seizure freedom rate of 70.8%, despite postoperative complications such as damage to adjacent corticospinal tracts, parietal dysfunction, partial-to-total aphasia if performed on the dominant side, and visual field defects [136]. PQD is a very effective and technically demanding procedure for clear-cut epileptic lesions of the posterior quadrant. However, it should not be performed on patients that exhibit subtle signs of pathology spreading into the whole hemisphere on pre-surgical MRI or seizures that propagate beyond the posterior quadrant (e.g., contralateral frontal lobe) [135]. Disconnective epilepsy surgeries are therefore suitable for static etiologies [133].

Frontal lobe disconnection (peri-insular anterior quadrantotomy) is used for extensive frontal epileptogenicity but inevitably disrupts fronto-striatal pathways [132]. Case reports show postoperative ipsilateral striatal neurodegeneration [132]. A retrospective study found that adjunctive disconnection of anterior thalamic and corticostriatal projections was associated with 3- and 5-year seizure freedom, suggesting that cortico-thalamostriatal networks may contribute to long-term epileptogenesis [137]. Late seizure recurrence has been reported to occur within 5 years postoperatively, unrelated to the extent of resection, suggesting that there may exist structures outside the EZ that generate new seizures [137].

Meta-analytic data indicate similar effectiveness and safety across lateral and vertical hemispheric disconnection approaches, with better outcomes in acquired/progressive vs. congenital/developmental etiologies [138]. Repeat resections and repeat disconnections show comparable rates of seizure control and postoperative complications, though temporary motor complications are more common after repeat resection [134]. Overall, disconnection can be preferred over resection due to lower morbidity, but choice of procedure must integrate etiology, seizure semiology, and patient-specific factors.

#### 3.2.3. Corpus Callosotomy

Palliative treatment of epilepsy is considered in cases of non-lesional seizure foci [139]. Corpus callosotomy (CC) is a palliative surgical procedure used for the treatment of intractable seizures of bilateral or diffuse origin or unilateral origin with progressive propagation to the contralateral cerebral hemisphere [140] (Figure 3). Patients with pharmacoresistant generalized (except for genetic generalized epilepsy) or multifocal epilepsy are candidates for CC, as focal resection is not feasible in these cases [141,142]. CC is particularly effective for drop attacks seen in childhood syndromes such as Lennox–Gastaut syndrome (LGS), West syndrome, and severe multifocal epilepsy [142,143]. The surgery is performed by means of severing the interhemispheric commissural white matter fibers, intercepting the interhemispheric spread of secondary generalized epileptic seizures [144]. Corpus callosotomy may include sectioning of the anterior two-thirds (anterior CC) or the entire corpus callosum (total CC) or occasionally the posterior structures (posterior CC) [141].

Evidence generally favors total over anterior CC for drop-attack control, although the former carries a higher risk of disconnection syndromes (split-brain syndromes) and language deficits [145]. One study reported greater reduction (*p* = 0.006) or elimination (*p* = 0.024) of drop seizures after total CC, and a meta-analysis confirmed that total CC more reliably prevents drop seizures [141,146]. Age of the patient and the duration of epilepsy prior to surgical intervention were found to not affect the surgery outcomes [141]. A study of pediatric epilepsy, however, reports no significant difference in seizure outcome between anterior and total CC [143]. Synchronization of fibers from the premotor cortex, supplementary sensorimotor area, and primary motor cortex that are localized to the posterior half of the corpus callosum was suggested to be specifically relevant to drop attacks [147]. In a study of the outcomes of selective posterior CC, the surgery allowed 83% of patients with drop attacks to achieve either complete or >90% control of the falls within at least 4-year follow-up [147]. As anterior callosal fibers are spared in posterior CC, lower risk of disconnection syndrome was suggested [145].

Other possible complications of CC include hydrocephalus, transient postoperative disturbance of consciousness, chemical meningitis, temporary postoperative hemiparesis, behavioral disturbances, and incidents related to craniotomy [145,148]. A recently reported case of postoperative anti-NMDAR encephalitis, which proved responsive to immunotherapy, may explain progressive neurological deficits following CC and suggests the need for preoperative antibody screening [148].

Minimally invasive CC techniques, including laser interstitial thermal therapy (LITT) and mini-craniotomy/endoscopic (mc-e) approaches, offer similar seizure outcomes to open surgery with fewer complications; LITT had no reported complications in a multicenter study [149]. Radiosurgical CC has also been explored, producing callosal axonal degeneration with favorable neuropsychological outcomes and comparable seizure control, but presenting possible complications such as symptomatic edema and rare radiation-induced malignancies [145,150].

A meta-analysis of 24 studies compared the outcomes of CC and a newer palliative surgical approach, vagus nerve stimulation (VNS), in pediatric patients [139]. The study found a statistically significant difference in the control of atonic seizures, favoring CC over VNS (*p* = 0.003), although CC resulted in a higher rate of complications and reoperations [139]. Another study, however, mentions severe complications of VNS such as sudden unexpected death in epilepsy (SUDEP), status epilepticus (SE), and vocal cord paralysis, favoring micro-surgical CC over VNS [150]. In general, research on seizure outcomes and complications of corpus callosotomy produced heterogeneous and debatable results, suggesting that there is an urgent need for extensive research to establish widely accepted outcome measures and guidelines for corpus callosotomy.

#### 3.2.4. Neuromodulation

Neuromodulation is a palliative treatment for patients with refractory epilepsy, who are deemed non-eligible for resective surgery, including those with epileptic activity in the eloquent cortex or when EZ cannot be localized on preoperative assessment [151] (Figure 3). Approved modalities include vagus nerve stimulation (VNS), deep brain stimulation of the anterior nucleus (ANT-DBS) and centromedian nucleus (CM-DBS) of the thalamus, and responsive neurostimulation (RNS) [152,153].

VNS acts on the vagus afferent network, presumably desynchronizing the epileptiform activity, modulating neurotransmitter release and thalamocortical circuits, or activating C-type fiber [151,154]. The device is typically implanted on the left vagus nerve, as stimulating the right vagus nerve could cause bradycardia due to its control over the sinoatrial node; however, right-sided VNS has been employed safely in pediatric patients [151,155]. A review of VNS registry data of 8423 patients suggests that VNS modifies neural circuits over time rather than temporarily suppressing seizure activity, with 50% responder rate (RR) reaching 63% and seizure freedom up to 8% in the long term [153,156]. Younger age, multifocal seizures, shorter epilepsy duration prior to VNS, seizure onset at temporal site, generalized epilepsy, and tuberous sclerosis have all been associated with better VNS response [151,154]. In addition to the known VNS complications such as infection at the site of implantation, hematoma, vocal cord paralysis, lower face weakness, and pain, there has been more evidence of de novo or worsening sleep breathing disorders [151,153]. The E03 and E05 studies, the largest randomized trials of VNS, reported that high-intensity VNS is more effective in seizure reduction than low-intensity VNS [151]. The closed-loop VNS, which launches the stimulation of vagus nerve following predefined ictal changes in the heart rate, has been reported to improve seizure control in 41% of cases compared to 31% of cases with standard VNS treatment [153].

ANT-DBS is effective in the management of focal and secondarily generalized forms of DRE [151]. In the Stimulation of the Anterior Nucleus of the Thalamus for Epilepsy (SANTE) trial, seizure reduction reached 75% at seven years, with infection (12.7%) and targeting errors (8.2%) at 10 years post-implant as common device-related complications [157]. The rate of SUDEP reported in this study was favorable (2.0 deaths per 1000 person-years), and the risk could further be reduced with long-term ANT-DBS therapy, similar to other neuromodulation methods (i.e., VNS, RNS) [157]. Reported seizure reduction varies widely (11.5–76%), likely due to differences in stimulation settings; high-frequency stimulation appears most beneficial [151,158]. ANT-DBS overrides pathological electrical activity, thereby indirectly modulating neuronal network excitability and inducing neuroprotective effects, causing decrease in neuronal cell loss, inhibition of immune response, and modulation of neuronal metabolism [158].

CM-DBS is effective for primary generalized DRE and Lennox–Gastaut syndrome [159]. In the Thalamic Centromedian Nucleus Stimulation for Lennox–Gastaut Syndrome (ESTEL) trial, 89% of patients receiving active stimulation achieved ≥50% electrographic seizure reduction, and 50% had similar reductions in diary-reported seizures [4]. A serious adverse effect included an infection with *Staphylococcus aureus* at the site of DBS implant, and other reported adverse effects such as food aversion/change in appetite, fatigue, headache, pain and discomfort, swallowing difficulty, and paresthesia were transient [4]. CM-DBS operates effectively at medium and high frequencies [151]. CM-DBS is proposed to act on reticulo-thalamo-cortical circuits, thereby desynchronizing epileptiform activity in the brain regions associated with LGS [151].

In comparison to open-loop paradigms such as VNS and DBS, closed-loop neurostimulation is well-suited to detect and abort only ongoing epileptiform seizures in the eloquent cortex [154]. NeuroPace RNS is the only commercially available and clinically approved closed-loop device [160]. Despite ongoing concerns among neuroethicists regarding the possibility that closed-loop neuromodulation systems may demonstrate a degree of “system autonomy”, evidence from a recent study challenges these apprehensions, presenting findings that suggest such effects may be less problematic than initially anticipated [160]. In the Pivotal trial, seizure reduction was significantly greater with RNS than sham stimulation (37.9% vs. 17.3%) [161]. A follow-up analysis of the Pivotal RCT demonstrated a median percent reduction in seizures of 44% at 1 year and 53% at 2 years, indicating a progressive and significant improvement post-implantation [162]. The incidence of infections at the site of RNS implantation has been reported at 4% per surgical intervention, with a cumulative infection rate affecting 12% of patients over a nine-year follow-up period [153]. Although closed-loop stimulation represents a promising advancement in neuromodulation, current evidence has not demonstrated its superiority over open-loop stimulation in terms of therapeutic efficacy [151].

In clinical practice, the choice between surgical approaches is primarily determined by the spatial organization of the epileptogenic network and its relationship to eloquent cortex. Evidence-based reviews outline the selection principles and the range of surgical and neuromodulation strategies available for drug-resistant epilepsy [163,164]. Resective approaches are generally preferred when a well-defined and focal epileptogenic zone can be safely removed, offering the highest likelihood of long-term seizure control. In contrast, disconnective procedures are considered when the epileptogenic network is extensive or multilobar and direct resection carry excessive risk of neurological deficit. Neuromodulation is typically reserved for patients in whom neither resection nor disconnection is feasible due to multifocality, poor localization, or eloquent cortex involvement, or when seizure reduction rather than cure is the primary goal. This clinical decision-making framework reflects a balance between seizure outcomes and preservation of neurological function based on individualized assessment.

## 4. New Anti-Epileptic Drug Trials

Numerous preclinical and clinical trials assessing the efficacy and safety profile of potential novel epilepsy drugs that target various underlying molecular mechanisms of epilepsy are underway. An exhaustive list of epilepsy drug candidates currently in development is summarized in the Supplementary Tables S2 and S3.

## 5. Novel Anti-Seizure Medications and Innovative Research

The management of epilepsy is rapidly evolving beyond established treatment strategies, driven by a surge in innovative research aimed at providing more effective and personalized care. This new frontier includes the exploration of herbal medicinal plants with anticonvulsant properties, alongside the development of diagnostic tools powered by artificial intelligence, machine learning, and deep learning to enhance detection and characterization of epileptogenic networks. Precision therapies, including gene-based interventions, metabolic modulation, and biomarker-guided treatments, represent a novel direction aimed at individualized care. In parallel, surgical innovations continue to evolve, offering less invasive and more targeted techniques that may improve outcomes for patients with drug-resistant epilepsy.

### 5.1. Promising Herbal Medicinal Plants

**Figure 4:**
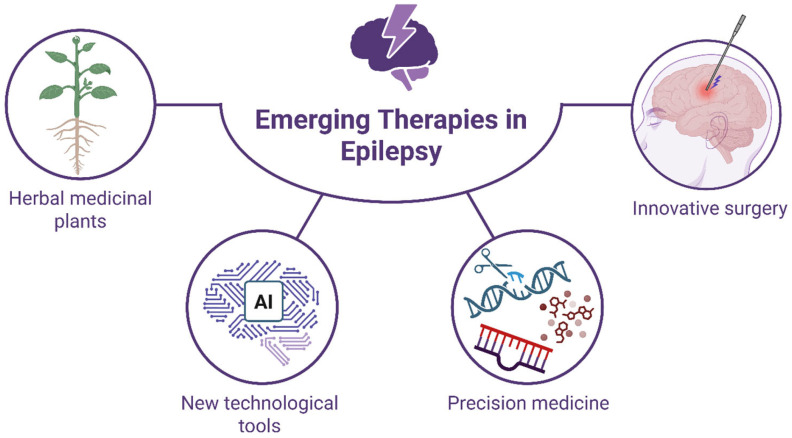
Emerging therapies in epilepsy. Summary of key areas of innovation in epilepsy treatment: (1) herbal medicinal plants under investigation; (2) AI, machine learning, and deep learning tools; (3) precision therapies (gene-based, metabolic, biomarker-guided); and (4) advances in surgical techniques.

In the ongoing search for more effective and safer treatments for epilepsy, researchers have increasingly turned their attention to herbal medicinal plants (Figure 4). This interest is driven by the limitations of current anti-epileptic drugs, which often fail to control seizures in a significant portion of patients and can cause undesirable side effects. Traditional medicine systems, such as those in China and Iran, have long utilized various plants to manage seizures, suggesting a wealth of therapeutic potential [165]. Scientific investigations have identified numerous plant-derived compounds with anticonvulsant properties, including flavonoids, alkaloids, and terpenoids, which may modulate neurotransmitter systems or ion channels involved in seizure activity. Several examples of promising anticonvulsant phytoconstituents are summarized in Supplementary Table S4, focusing on their possible molecular targets and effects generated (Supplementary Table S4). Compounds such as aconitine, piperine, ginsenosides, curcumin, resveratrol, quercetin, and luteolin have shown anticonvulsant or neuroprotective effects in rodent models, zebrafish larvae, or in vitro systems, manifested as delayed seizure onset, reduced seizure severity or frequency, and attenuation of seizure-induced neuronal damage [166,167,168,169,170,171]. In contrast, translation to human studies remains limited. Among the phytoconstituents listed, gastrodin, a phenolic constituent of Gastrodia elata, has been evaluated in clinical studies in combination with anti-seizure drugs and has shown some benefits in seizure control in controlled settings [172]. In addition, nanomicelle curcumin (a high-bioavailability formulation of curcumin) was investigated in a double-blinded, randomized crossover clinical trial of children with intractable epilepsy, where add-on nano-curcumin significantly reduced seizure frequency compared with placebo over a 10-week period, suggesting potential therapeutic benefit in pediatric refractory epilepsy [173]. However, these human studies are preliminary, involve relatively small cohorts, and require replication in larger, controlled trials to confirm efficacy and safety.

### 5.2. Emerging Diagnostic Tools for Epilepsy

Diagnosis of epilepsy can be established by patient history, videos of spells, neurophysiology (e.g., electroencephalography [EEG]), neuroimaging), and wearable device data [174]. Manual review of EEG and MRI is time-consuming, susceptible to artifacts, and can miss subtle abnormalities, motivating the development of automated models for detection of epileptiform activity based on artificial intelligence (AI), machine learning (ML), and deep learning (DL) [175,176] (Figure 4). Epileptologists using these systems report comparable accuracy to expert review but with significantly reduced interpretation time [174].

Classic ML algorithms such as support vector machines (SVMs) and k-nearest neighbor (k-NN) are used to identify abnormal EEG waveforms and epochs with minimal training data but require substantial manual labeling [177]. Convolutional neural networks (CNNs), a more intricate ML model that requires less pre-processing of the input data, have been used in processing and analysis of EEG [177]. The SpikeNet model, trained on 13,262 interictal epileptiform discharges (IEDs) from 9571 scalp EEG records, exceeded human expert performance with 67% sensitivity, 63% specificity, and 65% accuracy [178,179]. DeepSpike, a fast region-based CNN (R-CNN) model trained across heterogeneous EEG sources, achieved 89% sensitivity, 70% specificity, and 80% accuracy, correctly localizing focal and generalized epileptiform discharges [180]. An SVM algorithm, which has comparable performance to CNN models, has been used in multimodal wrist devices with sensitivities of 88–95% for tonic–clonic seizures, detection latencies of less than 40%, and false alarm rates of 0.2 per day [177].

ML tools can be used to detect seizures from sleep to decrease the variability of the control/false alarm events; ML analysis of video material showed a clear differentiation between hypermotor epileptic seizures and other sleep disorders [177,181]. ML-based analysis of seizure diaries and data obtained from wearable seizure detectors such as limb accelerometry (ACC), photoplethysmography, electrocardiography, and EEG can improve treatment efficacy and potentially provide seizure forecasting [174]. Hybrid CNN-RNN models trained on long-term scalp EEG can predict seizures up to 1 h before onset with 99.6% accuracy, and bilinear CNN-RNN frameworks have improved seizure-type classification [182,183]. Classification of focal and idiopathic generalized epilepsy in patients at age ≥10 years was achieved by a study that trained an ML algorithm on 1445 epilepsy patients, achieving precision of 81%, sensitivity of 81%, and specificity of 77% [184]. Another study aimed to detect a broad range of seizure types by developing DL-based seizure detection models [185]. The model utilized data from patients’ electrodermal activity, accelerometry, and photoplethysmography (for blood volume pulse (BVP)), with wearable recordings containing 900 seizures and 28 seizure types [185]. The fusion of ACC and BVP data with the CNN-long short-term memory (LSTM)-based seizure detection model demonstrated the best detection performance with sensitivity of 83.9% and false positive rate of 35.3% [185]. Moreover, ML tools can be used for identification of potential surgical candidates, improvement in surgical plans, and prediction of surgical outcomes [174].

Automated approaches can improve detection of subtle or MRI-negative pathologies such as hippocampal sclerosis (HS), which can otherwise hamper early diagnosis and surgical treatment [175]. An SVM classifier improved the detection rate of MRI-negative HS to 96.0% with the degree of gray-white matter boundary blurring in the temporal pole as the most important feature [186]. A metabolic-wise PET-based lateralization framework achieved 96.43% accuracy in detection of MRI-negative, PET-positive TLE [187]. Biomarkers such as high-frequency oscillation, interictal epileptiform discharge, and phase amplitude coupling obtained from interictal intracranial EEG recordings were used to develop an AI-based analytic framework for the localization of seizure onset zone [188]. Personalized brain network models, such as the Virtual Epileptic Patient (VEP), integrate multimodal non-invasive data of individual epilepsy patients to simulate individualized epileptic networks and guide intervention [189].

Importantly, the level of validation varies substantially across AI- and ML-based diagnostic studies. EEG detection models, including SpikeNet and DeepSpike, have been evaluated using large, independently annotated EEG datasets and benchmarked against expert consensus [178,179,180]. In contrast, seizure prediction, wearable-based detection, and classification models have been developed and tested primarily on retrospective or internally split datasets, often within single-center cohorts [182,183,184,185]. Similarly, AI approaches for MRI-negative epilepsy and seizure onset zone localization remain largely based on retrospective analyses or limited external validation [187,188]. Multicenter studies and real-world clinical validation are still limited and represent a critical requirement for translation into routine clinical practice.

Major barriers to clinical adoption include lack of large, high-quality, and diverse datasets, which limits model generalizability [177]. To complicate matters further, in case of EEG interpretation, there is not a reliable gold standard due to high inter- and intra-rater disagreement among EEG experts, and DL approaches require substantial computational resources [174,176]. It is important to note that there is a potential for ML/AI to perpetuate existing healthcare biases, as these rely on masses of publicly accessible information [174]. At the same time, these tools may not have access to important information contained in textbooks, peer-reviewed publications requiring subscription, and clinical datasets that are publicly unavailable [174,176]. Therefore, prospective and multicenter clinical trials are required to establish generalizability of these models, assess their cost-effectiveness, and compare them to existing practice [177].

### 5.3. Precision Therapy

The heterogeneity of epilepsy subtypes, their underlying mechanisms, individual factors, and pharmacokinetic variability of ASDs render the traditional treatment strategies ineffective for some patients [6]. The limitations of traditional approach in epilepsy treatment necessitate the urgent investigation of innovative therapies with a focus on personalized medicine for epilepsy patients (Figure 4).

#### 5.3.1. Gene-Based Therapy

Gene therapy is a precision medicine tool that has the potential to address the underlying genetic abnormalities in the context of epilepsy. The CRISPR/Cas9 system is a revolutionary genome editing approach that can be employed in the development of more targeted epilepsy treatments; however, it also brings significant obstacles related to its safe deployment in humans such as targeting specific regions of interest in the brain, triggering adaptive immune system to Cas9, and alleviating side effects at off-target sites [190,191]. The genome editing approach can be used to target genes associated with ion channels, neurotransmitter receptors, and signaling pathways that contribute to epileptogenesis [6]. Several preclinical studies have demonstrated reduced seizure frequency and improved seizure control in animal models with epilepsy-related genetic mutations subjected to gene therapy [6]. Another promising gene therapy tool is based on antisense oligonucleotides (ASOs), which correct or compensate for gain-of-function (GOF) and loss-of-function (LOF) genetic mutations by modifying expression of target mRNA by either altering mRNA splicing or promoting mRNA degradation [192]. ASOs are being tested in clinical trials of Dravet syndrome (a DEE), over 85% cases of which are found to have de novo LOF *SCN1A* variants, leading to Nav1.1 haploinsufficiency [193]. Non-coding regions of the *SCN1A* gene were found to enable inclusion of a poison exon, or nonsense-mediated decay exon, which reduces *SCN1A* gene expression, producing the phenotype of Dravet syndrome [194]. Targeted augmentation of nuclear gene output (TANGO) technology can target the mRNA transcript containing this poison exon, thereby decreasing its levels and boosting Nav1.1 protein production [193]. STK-001 is an ASO that entered clinical trials and utilizes the TANGO technology [193]. Both CRISPR-Cas9 systems and ASOs can use adeno-associated virus (AAV) vectors for controlled and targeted delivery of therapeutic genes, although AAVs have not yet been tested in humans in the contest of genetic epilepsy [6,191]. An original preclinical research has proposed a dCas9 (dead-Cas9) promoter-enhancing system packaged in an AAV vector, which aims to guide *SCN1A* gene regulation and proved to have substantial advantages for developing an effective and safe gene therapy strategy for Dravet syndrome in mouse model [195]. ETX-101 is a gene therapy delivered by an AAV-9, which expresses an engineered transcription factor to promote enhanced transcription and translation of the *SCN1A* gene in GABAergic interneurons, restoring their function [193]. Rigorous risk and benefit assessment, and ethical considerations are necessary to address the unique challenges of gene therapy approaches. Preclinical studies using gene therapy in animal models have shown promising results, and clinical trials involving human subjects with genetic epilepsies are underway [6]. Additionally, optogenetics have emerged as a groundbreaking technology that enables genetically engineered neurons to express proteins called opsins, which are activated in response to specific wavelengths of light and can either stimulate or inhibit activity of the targeted neurons [6]. This technology is in its early stages of development; however, it has the potential to inspire personalized and adaptive closed-loop neurostimulation systems when combined with RNS devices [196].

As gene-based therapies move closer to clinical implementation, ethical and regulatory challenges must be addressed. Safety concerns, including potential off-target effects and immune responses, necessitate rigorous preclinical testing and long-term monitoring beyond initial clinical endpoints [197,198]. Regulatory frameworks for gene therapy vary across jurisdictions, demanding strict oversight of trial design, vector manufacturing, and risk–benefit assessment, which can delay translation despite promising preclinical results [199]. Ethical considerations also include fair participant selection and justice in access, as high costs and limited availability of advanced gene therapies may exacerbate healthcare disparities, underscoring the need for policies that promote equitable implementation [200].

#### 5.3.2. Metabolic Therapy

The ketogenic diet (KD) is a high-fat, low-carbohydrate, and moderate-protein dietary approach, which can mimic favorable effects of fasting and has been known to reduce seizure frequency in some patients [6]. It is a standard therapy and clearly a precision medicine tool for the treatment of seizures secondary to GLUT1 deficiency [201]. GLUT-1 deficiency syndrome presents with high phenotypic variability that includes epilepsy and movement disorders, which can see improvement with the implementation of KD [190]. A study reported that following a treatment with KD, 95% of pediatric patients with GLUT1-deficiency syndrome had >50% seizure reduction and 80% of them had >90% seizure reduction [202]. Although the exact mechanisms of action of KD have not yet been elucidated, it is believed that the elevation in ketone bodies in the bloodstream can exert anticonvulsant properties and provide an alternative energy source for the brain, stabilizing neuronal function and dampening seizure activity [6]. KD may also exhibit neuroprotective and anti-inflammatory properties by enhancing mitochondrial function and biogenesis, abating oxidative stress, modifying the expression of potassium channels, enhancing the expression of the brain-derived neurotrophic factor (BDNF), facilitating purinergic and GABAergic neurotransmission, and modifying gut microbiota and thereby affecting the gut–brain axis [203]. The National Institute for Health and Care Excellence guidelines (NICE) underscored the effectiveness of KD in the treatment of multidrug-resistant epilepsy [203]. However, these effects of KD are out of the scope of precision medicine. In general, KD can be beneficial as complementary treatment for epilepsy and may offer improved seizure management.

#### 5.3.3. Biomarker-Guided Therapy

Pharmacological treatments for epilepsy target ictogenesis and are therefore classified as anti-seizure drugs, with no current therapies specifically designed to target epileptogenesis [204]. The search for genetic, molecular, cellular, imaging, and electrophysiological biomarkers for epileptogenesis is important for the identification of individuals who are more likely to develop epilepsy following an epileptogenic insult and could be enrolled in clinical trials of potential anti-epileptogenic drugs [205] (Figure 4). Biomarkers may also serve as predictive markers of treatment response, indicating the likely efficacy and safety of a specific anti-seizure drug in a given patient [205]. Ideal biomarkers should be non-invasive, stable, mechanistically informative, and compatible with economically feasible clinical trials and analysis platforms [204].

Some studies have shed light on potential biomarkers for epileptogenesis. Hair cortisol concentrations, measured within 24 h of a child’s first seizure, were significantly elevated compared to controls (7.5 pg/mg versus 5.0 pg/mg, respectively (*p* = 0.001)) suggesting sustained HPA axis activation that may contribute to seizure susceptibility and representing a prognostic biomarker of epileptogenesis [206]. High-frequency oscillations (80 Hz–500 Hz) have also been proposed as prognostic electrophysiological biomarkers [207]. Another study conducted whole genome analyses to test whether there was a difference in temporal cortical gene expression between seizure-free and non-seizure-free subjects following anterior temporal lobectomy with amygdalohippocampectomy (ATL/AH) [208]. The study associated relative down-regulation of four genes—zinc finger protein 852 (*ZNF852*), CUB domain-containing protein 2 (*CDCP2*), proline rich transmembrane protein 1 (*PRRT1*), and *FLJ41170* along with seven RNA probes—with seizure-free outcome following ATL/AH, representing predictive genetic biomarkers of response to surgery [208]. BBB dysfunction following TBI, detected by the leakage of gadolinium-based contrast agent resulting in an increase in the MRI T1 signal, can potentially serve as a prognostic biomarker for TBI-induced PTE [209]. Other emerging prognostic protein biomarkers for PTE are glial fibrillary acidic protein (GFAP), S100 calcium-binding protein B (S100B), IL-6, HMGB1, and ubiquitin C-terminal hydrolase L1 (UCH-L1), which require further studies to confirm their clinical significance [207].

### 5.4. Innovative Surgery

Epilepsy surgery has traditionally relied on resective procedures to remove seizure foci; however, these carry risks of neurological and surgical complications [210]. Advances in neuroimaging and minimally invasive technologies have enabled safer, more precise alternatives [210] (Figure 4).

MRI-guided laser interstitial thermotherapy (LITT) is a minimally invasive surgical technique used to treat focal epilepsies [211]. The technique employs stereotactic methods for accurate and precise placement of a laser probe into a therapeutic target, time-dependent laser ablation, and a real-time MRI thermographic monitoring [212]. LITT has been used to treat epileptic conditions such as mesial temporal sclerosis (MTS), hypothalamic hamartoma (HH), periventricular nodular heterotopia (PNH), focal cortical dysplasia (FCD), tuberous sclerosis, and cavernous malformations, with visual field defects as the most common side effect [213]. LITT offers a low overall complication rate and rapid recovery [214]. Following an LITT procedure, out of 71 HH patients, 93% were free of gelastic seizures at 12 months, but 23% of the patients had to undergo laser ablation more than once [215]. Reported seizure freedom rates of LITT for MTLE range between 36% and 78% at minimum of 1 year of follow-up [216]. In a multicenter retrospective cohort study comprising 234 patients, 58.0% of patients achieved Engel class I outcome at both 1 and 2 years following LITT [216]. Seizure freedom improves when intraoperative MRI thermography identifies residual focal cortical dysplasia and prompts additional ablation (73% vs. 38% Engel IA; *p* < 0.05) [214].

Stereoelectroencephalography-guided radiofrequency thermocoagulation (SEEG-RFTC) is a minimally invasive brain lesioning method, during which patients do not require general anesthesia, allowing for clinical monitoring [213]. In a meta-analysis comparing LITT, SEEG-RFTC, anterior temporal lobe resection (ATL), and selective amygdalohippocampectomy (sAHE), Engel Class I outcomes were 44% for SEEG-RFTC, 57% for LITT, 69% for ATL, and 66% for sAHE [217]. While LITT and SEEG-RFTC yielded similar seizure outcomes, both were inferior to ATL and sAHE at ≥60 months of follow-up [217]. Recovery after SEEG-RFTC is rapid, though transient complications may include hyponatremia, hyperphagia, hyperthermia, impaired consciousness, short-term memory loss, Horner syndrome, weight gain, and hemorrhage [211,218].

Gamma knife (GK) is another non-invasive method that directs convergent small gamma rays on the EZ, sparing surrounding tissues with minimal radiation [211]. Radiation-induced necrosis at the EZ may develop gradually, occurring months to years after the procedure [211]. A total of 60–75% of patients with MTLE who underwent GK achieved seizure freedom at long-term follow-up, which is comparable to the results of microsurgery [219]. Although GK has lower complication rates compared to microsurgery, the delay in seizure improvement following GK can cause major morbidity and mortality [219]. Although there has been some concern regarding the potential for radiosurgery to induce tumor formation, current evidence has not established a definitive causal link between the procedure and the development of new neoplasms [211].

MRI-guided high-intensity focused ultrasonography (MRI-HIFU) enables non-invasive ablation of deep or cortical targets, including the anterior thalamus, amygdala, hippocampus, piriform cortex, and lesional pathologies such as FCD, PNH, and HH, using high-frequency waves to generate frictional heat exceeding 56 °C to induce cell death [213]. Real-time MRI monitoring minimizes off-target damage [211]. Possible side effects include skin burns at the site of ablation, damage to the dura and nearby brain parenchyma due to insufficient cooling, and bleeding risk [211]. There have been case reports from Japan utilizing HIFU to treat MTS and HH [119]. Case reports suggest that HIFU is a promising minimally invasive technique for treating intractable epilepsy; however, more studies are needed to assess the efficacy and safety of this method in treatment of epilepsy [119].

## 6. Discussion

Despite decades of research and clinical progress, epilepsy remains a challenging neurological disorder with considerable variability in its presentation, etiology, and response to treatment. The etiological complexity, which includes structural, genetic, metabolic, and inflammatory causes, often complicates treatment strategies, especially in drug-resistant epilepsy (DRE), which affects up to 40% of patients [4]. While over 25 anti-seizure drugs (ASDs) have been approved, and several more are under development, long-term seizure control remains elusive for many individuals [3]. This review evaluates the translational relevance and clinical impact of recent advances in surgical innovations, genetic screening, and emerging technologies such as AI, ML, and precision medicine. Together, these developments are opening new avenues for improved diagnosis, treatment, and patient outcomes in epilepsy. At the same time, each approach presents unique challenges and limitations.

Surgical techniques, from traditional resections to minimally invasive approaches like laser interstitial thermal therapy (LITT) and gamma knife radiosurgery, have improved seizure control in selected patient populations [7]. Functional disconnection surgeries, including corpus callosotomy and hemispherotomy, offer palliative solutions for patients with multifocal or generalized seizures. However, complications such as visual field defects, neuropsychological impairments, and postoperative seizure recurrence limit their widespread applications [136,137]. Neuromodulation methods, including VNS, ANT-DBS, CM-DBS, and RNS, are increasingly used for patients who are not candidates for resection. Though these techniques provide significant seizure reduction, their therapeutic efficacy varies, and long-term outcomes still require further investigation and validation [151,153].

Precision medicine has added a transformative layer to epilepsy treatment, particularly regarding genetic epilepsies. From a diagnostic perspective, trio-based sequencing and high-resolution genomic platforms have significantly increased the detection of pathogenic variants, enabling genetic confirmation in disorders that previously remained idiopathic [9,14]. In generalized epilepsies, GWAS discoveries - including loci at CACNA2D2, SCN8A, and BCL11A - offer clinically actionable biomarkers that may assist in early risk prediction, inform diagnostic algorithms, and refine phenotypic classification [13,14]. These genetic signals also highlight ion channel and synaptic pathways already targeted by existing anti-seizure medications, underscoring opportunities for therapeutic repurposing. In focal epilepsy, recognition of mTOR pathway dysregulation, whether germline (e.g., *DEPDC5*, *NPRL2*, *NPRL3*) or somatic (e.g., *AKT3*, *PIK3CA*, *MTOR* variants), has immediate translational value. mTOR inhibitors, such as everolimus, already approved for tuberous sclerosis complex-associated seizures, represent one of the clearest examples of genotype-guided therapy in epilepsy [21]. Similarly, discovery of brain-restricted mosaic variants in *SLC35A2*, *KRAS*, and *BRAF* supports the use of lesion-specific profiling to inform prognosis and, potentially, advocate for targeted pathway inhibitors as adjunctive therapies in refractory cases. The finding that many pathogenic mosaic variants are undetectable in blood highlights the need for integrating advanced tissue-based diagnostics (e.g., ultra-deep sequencing of surgical specimens) into the clinical workflow, particularly for patients undergoing epilepsy surgery.

Insights into metabolic disturbances - altered glucose utilization, mitochondrial impairment, and inborn metabolic errors - inform diagnostic and therapeutic strategies. Characteristic patterns such as ictal hypermetabolism and interictal hypometabolism are now routinely detected through FDG-PET and can assist in localizing epileptogenic zones in focal epilepsy [29]. GLUT1 deficiency syndrome can be identified through SLC2A1 sequencing and corrected with ketogenic diet therapy [42,44,45], which shows a substantial translational impact. Detection of mitochondrial dysfunction through biochemical assays, muscle or fibroblast studies, or next-generation sequencing can guide prognosis and therapeutic decisions, including mitochondrial support strategies and avoidance of medications that exacerbate oxidative stress [34,51,54,56,57,58,59].

Understanding of inflammatory pathways and circulating molecular biomarkers would help to shape the clinical approaches to epilepsy. In particular, the HMGB1-TLR4/RAGE axis represents a pathway with potential translational relevance, supporting this pathway as promising therapeutic targets in epilepsy [65,66,67]. The ATP-P2X7R-NLRP3 pathway also provides a mechanistic basis for targeting purinergic receptors or inflammasome components to limit neuroinflammation and seizure propagation [68,71]. Cytokines such as IL-1β, IL-6, and TNF-α present clinical implications as both biomarkers and therapeutic targets. Elevated IL-1β contributes to BBB dysfunction, mTOR activation, and increased glutamatergic signaling - mechanisms that may underlie pharmacoresistant epilepsy [78,82,83,85]. These pathways have motivated early translational efforts using IL-1R antagonists. Likewise, dysregulated TNF-α signaling, with its receptor-dependent pro- and anticonvulsant effects, highlights the potential for selective cytokine-modulating therapies [91,92,93]. Inflammatory chemokines and complement activation also hold diagnostic relevance. Upregulation of CCL2, CCL3, and CCL4 in temporal lobe epilepsy, along with elevated serum C1q and iC3b in drug-resistant epilepsy, suggests that peripheral inflammatory profiling may assist in patient stratification [94,97]. Complement-driven synaptic remodeling represents another promising therapeutic target [79,80]. Emerging molecular biomarkers further strengthen the translational pipeline. Circulating tRNA fragments detectable before seizure onset represent potential tools for early diagnosis and monitoring [98]. In addition, microRNA-146a and other inflammation-linked microRNAs consistently show altered expression in patients with epilepsy and may serve as accessible biomarkers for risk prediction and disease progression [99,100,102,103]. However, it should be noted that no biomarker has yet been introduced into clinical practice due to the presence of practical hurdles. A major challenge is limited reproducibility across independent cohorts, reflecting the marked biological heterogeneity of epilepsy with respect to etiology, seizure networks, comorbidities, and treatment history, which complicates cross-study validation [220,221]. In addition, many biomarkers exhibit state-dependent variability, with levels fluctuating during different seizure phases or disease stages, complicating standardization and clinical interpretation [222]. Moreover, most proposed biomarkers remain in the discovery or early validation phase, lacking prospective longitudinal studies, predefined clinical thresholds, and large multicenter replication cohorts required for regulatory qualification and clinical implementation [223]. Collectively, these challenges help explain the enduring gap between biomarker discovery and clinical translation in epilepsy and underscore the need for harmonized study designs, rigorous validation frameworks, and clearly defined clinical contexts of use.

In addition to molecular, metabolic, and inflammatory mechanisms, disruptions in functional brain connectivity represent a key dimension of epilepsy pathophysiology that helps explain cognitive comorbidities. Early multimodal imaging work demonstrated widespread network alterations in chronic epilepsy with significant associations with cognitive impairment [104]. These functional connectivity alterations appear to reflect both loss of network integrity within cognitive systems and compensatory changes that may not fully preserve performance. Integrating functional connectivity assessments with genetic, metabolic, and inflammatory biomarkers can provide a more comprehensive framework for understanding cognitive dysfunction in epilepsy and inform approaches such as network-guided neuromodulation or cognitive rehabilitation to improve network resilience and cognitive outcomes.

Interest in herbal medicine has been rejuvenated, with compounds such as ginsenoside Rg3 from Panax ginseng and tetrandrine from *Stephania tetrandra* demonstrating anticonvulsant properties via modulation of calcium influx and neuronal excitability [169]. While preclinical findings are encouraging, more rigorous clinical trials are needed to establish safety and efficacy. The role of herbal medicine, though historically underexplored, presents a rich area for future pharmacological innovation. Standardizing extraction methods, understanding pharmacokinetics, and conducting double-blind randomized controlled trials will be essential steps toward integrating herbal compounds into mainstream epilepsy care.

Looking forward, large-scale multicenter trials are essential to validate candidate biomarkers and develop reliable tools for early detection and individualized therapy. Emphasis should also be placed on integrating genetic, metabolic, and inflammatory markers into a cohesive diagnostic framework that can stratify patients based on risk and therapeutic responsiveness. In summary, epilepsy research and treatment have entered a transformative era marked by innovations in molecular genetics, neuroimaging, surgery, and computational science. While significant strides have been made, major gaps remain, particularly in the management of drug-resistant and idiopathic forms. The future of epilepsy care lies in a personalized, mechanism-driven approach that unites clinical acumen with cutting-edge technology and multidisciplinary collaboration. A continued focus on translational research, patient-centered outcomes, and global accessibility will be key in making the promise of precision medicine a reality for all individuals living with epilepsy.

## Data Availability

No new data were created or analyzed in this study. Data sharing is not applicable to this article.

## Supplementary Materials

The following supporting information can be downloaded at https://www.mdpi.com/article/10.3390/ijms27031175/s1.
