# Supplementary material for: Epileptogenesis and Epilepsy Treatment: Advances in Mechanistic Understanding, Therapeutic Approaches, and Future Perspectives
Source: Int J Mol Sci. 2026 Jan 23;27(3):1175. doi: 10.3390/ijms27031175 (PMC12897611; doi:10.3390/ijms27031175)
Supplement: Supplementary file 1 [file ijms-27-01175-s001.zip › ijms-4099683-supplementary.pdf]

Supplementary Tables

Supplementary Table S1. Three Generations of ASDs: An Overview of Mechanisms, Clinical Applications, and Adverse Effects.

Anti-seizure drugs	Year of Introduction	Possible mechanism (s) of action	Clinical indications	Side effect profile	References
First generation					
Phenobarbital	1910	A positive allosteric modulator of $GABA_A$ receptor; antagonist of glutamate AMPA receptors; blocks high-voltage activated family of voltage-dependent calcium channels (L- and N-type calcium channels)	First-line therapy of choice for the management of neonatal seizures; second-line therapy for focal-onset and generalized tonic-clonic seizures	Intravenous phenobarbital can cause severe sedation, respiratory depression, and hypotension due to a long half-life; teratogenicity (congenital malformations – cardiac defects); hepatotoxicity; can cause osteoporosis if chronically used	[1–5]
Phenytoin	1940	Increases fast inactivation of voltage-gated sodium channels; blocks persistent sodium currents; blocks high-voltage activated family of voltage-dependent calcium channels	Second-line therapy for focal-onset and generalized tonic-clonic seizures	Gingival hyperplasia (60%), hirsutism, osteopenia/osteoporosis, cardiac arrhythmia (0.8%), Stevens-Johnson syndrome (SJS) (1-10 per 10,000 new users); teratogenicity (fetal hydantoin syndrome); hepatotoxicity	[1–6]
Ethosuximide	1958	Blocks low-voltage activated T-type calcium channels	First-line therapy of choice for absence seizures	Psychiatric adverse effects (depression, irritability, psychotic symptoms), SJS	[1–5]
Valproate	1960	Inhibits voltage-gated sodium channels, inhibits GABA transaminase and succinate semialdehyde dehydrogenase – GABA potentiation	First-line therapy for generalized tonic-clonic seizures, myoclonic seizures, absence seizures, and unclassified seizures; second-line therapy for focal-onset seizures; Lennox–Gastaut syndrome, infantile spasms (West syndrome), Dravet syndrome	Teratogenicity (neurocognitive deficits); hepatotoxicity; can cause osteoporosis if chronically used; can exacerbate tremor; possible thrombocytopenia/ neutropenia (1%-30%), weight gain (< 70%), osteopenia/ osteoporosis, pancreatitis (1 in 3,000 to 1 in 5,000 cases)	[1–6]

Carbamazepine	1974	Increases fast inactivation of voltage-gated sodium channels; blocks persistent sodium currents	Second-line therapy for focal-onset and generalized tonic-clonic seizures	Hyponatremia (1%-40%), neutropenia, osteopenia/ osteoporosis, SJS (1-10 per 10 000 new users)	[1–3,5,6]
Clonazepam	1974	A positive allosteric modulator of $GABA_A$ receptor.	Second-line therapy for myoclonic and absence seizures.	Sedation and tolerance	[1–5]
Clobazam	1979	A positive allosteric modulator at the $GABA_A$ receptor	Second-line therapy for generalized tonic-clonic seizures and third-line therapy for focal-onset seizures	Sedation and hepatotoxicity	[1–5]
Second generation					
Vigabatrin	1989	Inhibits GABA transaminase – GABA potentiation	Third-line therapy for focal-onset seizures; especially effective for refractory focal seizures and infantile spasms (West syndrome)	Weight gain, edema, increased irritability, elevated blood pressure, heart failure, blood sugar irregularities, increased risk of infection and kidney calcification (if combined with steroids/hormones), and visual field defects due to retinal toxicity (28.6%)	[1,3–6]
Oxcarbazepine	1990	Increases fast inactivation of voltage-gated sodium channels; blocks persistent sodium currents	Second-line therapy for focal-onset seizures; partial seizures	Hyponatremia (1%-46%), osteopenia/ osteoporosis, SJS;<br>may cause memory and concentration problems, and syndrome of inappropriate antidiuretic hormone secretion	[1–6]
Lamotrigine	1991	Increases fast inactivation of voltage-gated sodium channels; blocks persistent sodium currents; has some effect on GABA potentiation	First-line therapy for focal-onset seizures; second-line therapy for generalized tonic-clonic seizures, myoclonic seizures, absence and unclassified seizures;	Moderate ataxia, bipolar triggering, hypersensitivity and multi-organ failure with chronic usage; possible hepatotoxicity, teratogenicity, and pancreatitis; SJS (1-10 per 10,000 new users)	[1–6]

			effective in Lennox-Gastaut syndrome		
Gabapentin	1993	Modulates presynaptic release machinery via $\alpha 2\delta$ subunit of voltage-gated calcium channels; blocks persistent sodium currents	Second-line therapy for focal-onset seizures	Gabapentin-induced hypoglycemia, psychological and behavioral problems (anxiety/agitation and depression); weight gain (2%-3%), peripheral edema (2%-8%); ataxia, tremor	[1–6]
Felbamate	1993	Allosteric modulator of $GABA_A$ receptor; antagonist of NMDA receptor	Third-line therapy for focal-onset seizures; partial seizures and Lennox-Gastaut syndrome	Loss of appetite, insomnia, aplastic anemia, liver failure	[1–6]
Topiramate	1995	Blocks persistent sodium currents; antagonist of NMDA receptor; inhibits carbonic anhydrase; increases fast activation of voltage-gated sodium channels	Second-line therapy for focal-onset seizures, generalized tonic-clonic seizures, myoclonic and unclassified seizures	Weight loss (4%-17%), memory problems and verbal dysfunction (1%-11%), nephrolithiasis (3%); paresthesia, gastrointestinal problems, hyperammonemia	[1–6]
Tiagabine	1997	Inhibits GAT1 GABA transporter	Third-line therapy for focal-onset seizures	Cognitive side effects (memory loss, depression), tremor, possible stupor, hallucination, tachycardia, and elevated blood pressure	[1,3–6]

Levetiracetam	2000	Blocks high-voltage activated voltage-gated calcium channels; antagonist of AMPA receptor; modulates presynaptic release machinery by binding to synaptic vesicle glycoprotein 2A (SV2A)	Second-line therapy for focal-onset seizures, generalized tonic-clonic seizures, myoclonic seizures, absence and unclassified seizures	Psychiatric symptoms (7%-25%): irritability, hostility, depression, and hyperarousal; possible ataxia and confusion with high doses	[1–6]
Zonisamide	2000	Inhibits carbonic anhydrase; increases fast activation of voltage-gated sodium channels	Second-line therapy for focal-onset seizures, generalized tonic-clonic seizures, myoclonic seizures, and unclassified seizures	Psychiatric symptoms (1%-9%): psychosis, irritability, aggression, and major depression; memory problems and verbal dysfunction (10%-15%), SJS, nephrolithiasis (4%); may trigger anorexia, weight loss, vertigo, reduced sweating, and pancreatitis	[1–6]
Third generation					
Pregabalin	2004	Inhibits $\alpha 2\delta$ subunit-containing voltage-dependent calcium channels	Third-line therapy for focal-onset seizures	Weight gain, peripheral edema, dry mouth, ataxia, tremor	[1–5]
Lacosamide	2007	Increase of slow inactivation of voltage-gated sodium channels; block of persistent sodium currents	Second-line therapy for focal-onset seizures	Diplopia, cardiovascular abnormalities, skin rash, hematotoxicity, psychological symptoms (suicide risk)	[1–6]
Rufinamide	2008	Increases fast inactivation of voltage-gated Na+ channels; stimulates calcium-activated potassium currents while inhibiting sodium currents	Third-line therapy for focal-onset seizures; Lennox-Gastaut syndrome	Leukopenia, cardiac conduction (QT interval shortening), ataxia, diplopia	[1,3–6]

Eslicarbazepine	2009	Increases fast inactivation of voltage-gated Na+ channels; blocks persistent sodium currents; blocks low-voltage activated T-type calcium channels	Second-line therapy for focal-onset seizures	Hyponatremia (1%-2%), SJS; ataxia, tremor, blurred vision, diplopia; nasopharyngitis	[1–6]
Perampanel	2012	Antagonist of AMPA receptor	Second-line therapy for focal-onset seizures and generalized tonic-clonic seizures	Psychiatric adverse effects (depression, anxiety, impulsive behavior); weight gain, gait disturbance	[1,3–6]
Brivaracetam	2016	Modulates presynaptic release machinery by binding to synaptic vesicle glycoprotein 2A (SV2A)	Second-line therapy for focal-onset seizures, generalized tonic-clonic seizures, and myoclonic seizures	Teratogenicity (embryo death); abnormal coordination, nystagmus, mood changes; possible hypersensitivity	[1,3–6]
Everolimus	2018	Inhibitor of the mTORC1	Tuberous sclerosis complex-associated focal-onset seizures	Stomatitis, pyrexia, pneumonia, diarrhea, hypercholesterolemia, possible hypersensitivity, infections, renal toxicity, myelosuppression	[1,3–5,7]
Stiripentol	2018	A positive allosteric modulator of $GABA_A$ receptor	Adjunctive therapy for childhood epilepsy syndrome known as severe myoclonic epilepsy in infancy or Dravet syndrome	Anorexia, weight loss, imbalance, and hyperammonemia encephalopathy	[1,3–6]
Cannabidiol	2018	Blocks persistent sodium currents; acts on G-protein coupled receptor GPR55, transient receptor potential cation channel TRPV1, voltage-gated sodium channels, and equilibrative nucleoside transporter ENT1	Dravet syndrome, Lennox-Gastaut syndrome, tuberous sclerosis complex	Anorexia, diarrhea, sleep disorders, behavioral disturbances; possible hypersensitivity, hepatotoxicity, and pneumonia	[1,3–5]

Cenobamate	2019	Blocks persistent sodium currents; allosteric modulator of $GABA_A$ receptor	Third-line therapy for focal-onset seizures	Hyperkalemia, QT shortening, balance disorder, diplopia; nystagmus, drug eruption with eosinophilia and systemic symptoms (DRESS); possible hypersensitivity	[1,3–6]
Fenfluramine	2020	Agonist of serotonergic 5-HT2 receptor and antagonist of σ1 receptor	Dravet's syndrome; Lennox-Gastaut syndrome	Anorexia, decreased weight, diarrhea, constipation, coordination disturbances, behavioral disturbances, possible cardiac valvulopathy, pulmonary hypertension, serotonin syndrome	[1,3,4]

Supplementary Table S2. Preclinical Profile of Investigational Anti-epileptic Drugs.

Names	Mechanism(s)	Clinical indications	Sponsor	Final outcomes	References
ETX-123	Kv7.2/3 channel opener	Refractory focal-onset and generalized epilepsy	Eliem Therapeutics	No peer-reviewed publications are available. According to a company report, initial *in vivo* preclinical data for ETX-123 show a 7-fold safety margin between its effective dose for preventing tonic convulsions in the rat MES model (2 mg/kg) and the dose causing CNS side effects in the rotarod model (14 mg/kg).	[8,9]
NMT-001	Inhibits miR-134, which appears upregulated in experimental models of status epilepticus	Refractory epilepsy	NEUmiRNA Therapeutics	In a proof-of-concept screen using intra-amygdala kainate mouse models of mesial temporal lobe epilepsy, NMT-001 significantly reduced lorazepam-resistant status epilepticus seizures.	[9]
CAP-002	AAV vector-based product for syntaxin-binding protein 1 (STXBP1) mutations	STXBP1 DEE (developmental and epileptic encephalopathy)	Capsida Biotherapeutics	Preclinical studies in mice showed that intravenous delivery of a capsid carrying STXBP1 led to dose-dependent improvements in spike-wave discharges and cognitive and motor deficits, with effects lasting at least 12 months. In non-human primates, IV administration achieved broad brain transduction	[9]

				(neocortex, caudate, putamen, thalamus) while sparing the liver.	
GAO-3-02	Inhibits the cannabinoid type 2 (CB2) receptor	Generalized clonic seizures	GAOMA Therapeutics	GAO-3-02 significantly reduced seizure frequency and severity in several models—including PTZ, pilocarpine-induced epilepsy, and fully kindled rats—and restored cognitive function, with its antiseizure effects mediated via CB2 receptor activation. These benefits persisted up to a week after treatment.	[10]
LRP-661	May share same molecular targets as cannabidiol	Lennox– Gastaut syndrome; Dravet syndrome; tuberous sclerosis complex	London Research & Pharmaceuticals	LRP-661 significantly reduced seizures in multiple rodent models (MES, 6-Hz, PTZ, kindling, and corneal kindling), lacked THC-like behavioral side effects, and showed a broad-spectrum antiseizure profile, including in models predictive of efficacy in Dravet and Lennox–Gastaut syndromes.	[10]
FV-009	Selectively inhibits P/Q-type calcium channels (Cav2.1/2.2) and Nav1.7	Focal and generalized seizures	Trillium Therapeutics	FV-009 demonstrated strong efficacy in MES, PTZ, 6-Hz, AGS, and kindling models, with a therapeutic index ≥15 and robust activity in both epilepsy and pain models.	[11]
FV-082	Interacts with human recombinant enzyme monoamine oxidase B and inhibits its voltage-gated Nav1.8 sodium channels	Focal and generalized seizures	Trillium Therapeutics	FV-082 exhibited potent antiseizure activity in MES, 6-Hz, AGS, corneal kindling, and limbic seizure models, along with excellent safety (TD_{50} >600 mg/kg; therapeutic index ≥34) and broad oral bioavailability. It showed utility in pharmacoresistant epilepsy and pain models.	[3,12]
FV-137	Inhibits P/Q type Cav2.1/ β4/ α2δ1 and Cav2.2/ β3/ α2δ1 calcium channels; inhibits voltage-gated Nav1.6 and Nav1.7 sodium channels	Focal and generalized seizures	Trillium Therapeutics	FV-137 showed broad-spectrum efficacy across MES, PTZ, 6-Hz, AGS, and multiple kindling models, including lamotrigine-resistant seizures, with a favorable safety margin (max tolerated dose >500 mg/kg).	[3,13]

Losartan	Inhibits TGFβ signaling; blocks the increase in phosphophorylated Smad 2/3 levels following BBB disruption; neuroprotective	Insult-related epilepsies; TLE	-	Losartan inhibits albumin-induced TGF-β activation in the brain and prevents the development of delayed recurrent spontaneous seizures in rat models of vascular injury, with lasting effects post-treatment. These findings support losartan as a potential anti-epileptogenic therapy for epilepsy linked to vascular injury.	[14,15]
Fingolimod	Anti-inflammatory; prevents leukocyte infiltration into the brain parenchyma; blocks sphingosine-1-phosphate receptors with broad action at the CNS	Genetic absence epilepsy, Lafora disease (myoclonic seizures)	-	Early long-term treatment with fingolimod (1 mg/kg/day) before seizure onset in WAG/Rij rats showed temporary anti-epileptogenic and antidepressant-like effects, which faded five months after discontinuation. These effects were linked to transient suppression of mTOR signaling. Notably, fingolimod had more sustained benefits on cognitive function, associated with increased histone H4K8 acetylation. Overall, fingolimod shows promise in delaying epileptogenesis and cognitive decline, though its seizure-related effects may not be lasting. Additionally, FGD showed beneficial effects in a mouse model of Lafora disease by reducing astrocyte-driven neuroinflammation and T-cell infiltration, which was associated with improved behavioral outcomes.	[15–17]
Metformin	AMPK-dependent and AMPK-independent mechanisms; anti-inflammatory	TLE, Lafora disease (myoclonic seizures)	-	Metformin treatment preserved neuronal structure and reduced cell damage in lithium-pilocarpine model of SE in rats, demonstrating neuroprotective effects against TLE-related neuronal death. These benefits are attributed to AMPK activation, which promotes energy balance, inhibits apoptosis and JNK hyperactivation, and supports neurogenesis. Metformin also reduced elevated proinflammatory markers (IL-1β, NF-κβ, COX-2), microglial activation (CD11b), and astrogliosis (GFAP) in key brain regions.	[17,18]

				Behaviorally, metformin improved spatial learning and memory, as reflected in enhanced performance in maze tests.<br>In a mouse model of Lafora disease, metformin reduced key pathological features—such as polyglucosan and polyubiquitin aggregates and reactive astrogliosis—leading to improved neuropsychological outcomes. It also decreased seizure susceptibility, frequency, and duration, and prevented PTZ-induced mortality.	
Isoflurane	Induces an epigenetic downregulation of brain-derived neurotrophic factor receptors and tyrosine receptor kinase B; induces GABA potentiation; inhibits NMDA-gated and voltage-gated calcium currents; reduces synaptic glutamate release; neuroprotective and anti-inflammatory	Status epilepticus	-	Isoflurane's effects were studied in two SE-induced epilepsy rat models: intrahippocampal kainate and systemic paraoxon administration. Without isoflurane, most rats in both models developed frequent spontaneous recurrent seizures. Isoflurane administered during kainate injection did not alter SE duration or severity but significantly reduced the incidence of spontaneous seizures. A similar anti-epileptogenic effect was observed when isoflurane was given after SE in paraoxon-treated rats. In this model, isoflurane also prevented BBB disruption and neurodegeneration, while in the kainate model, it reduced neuroinflammation.	[15,19]
Anakinra	Blocks the action of IL-1α and IL-1β; anti-inflammatory	Status epilepticus, TLE	-	Anakinra and lamotrigine, whether alone or combined, significantly reduced neuronal loss in the CA1 region of the hippocampus in lithium–pilocarpine model of TLE in rats. In the CA3 region, monotherapies were more effective than combination treatment. While the therapies improved activity, exploration, and anxiety, they had limited impact on TLE-related social and memory deficits.	[15,20]
Atorvastatin	Inhibits the enzyme HMG-CoA reductase	Status epilepticus	-	Atorvastatin had no effect on SE duration or epilepsy development in rat model of TLE. Six weeks post-SE, BBB leakage persisted in limbic	[15,21]

				regions in both treated and control rats. Atorvastatin did not reduce inflammation, neuronal loss, or synaptic reorganization.	
Ceftriaxone	Increases EAAT2 (human excitatory amino acid transporter 2) expression via the NFκB signaling pathway	TBI-induced posttraumatic epilepsy (PTE)	-	In a mouse model of TBI, delayed ceftriaxone treatment (200 mg/kg/day from day 55 to 70 post-injury) upregulated glutamate transporters (GLT-1/GLAST) and significantly reduced severity, duration, and mortality of PTZ-induced seizures, even when administered weeks after TBI onset.	[15,22]
Rapamycin	Inhibitor of mTOR	Status epilepticus; TLE; TBI-induced PTE	-	In a $FeCl_2$-induced PTE rat model, rapamycin treatment significantly reduced behavioral seizure frequency, mitigated epileptic brain injury, and downregulated activated mTOR pathway markers (p-mTOR, p-P70S6K), indicating strong anti-epileptogenic and neuroprotective effects.	[15,23]
Dimethyl Fumarate	Activates the nuclear factor erythroid 2-related factor (Nrf2) - antioxidant; reduces microglial activation (Iba1) and the infiltration of CD4+ and CD8+ T-lymphocytes in the brain; activates HCAR2 - anti-inflammatory	Status epilepticus	-	In the kainic acid-induced SE model of TLE in rats, DMF treatment activated Nrf2 signaling, reduced neuronal death, decreased frequency and total number of spontaneous seizures, prevented epilepsy development in 3/10 animals, and reversed behavioral deficits, with seizure reduction lasting weeks after cessation.	[17,24]
Propranolol	Ameliorates microglial reactivity – anti-inflammatory, neuroprotective	Lafora disease (myoclonic seizures)	-	Chronic propranolol treatment significantly improved attention deficits and restored neuronal organization in the hippocampus of Lafora ($Epm2b^{-}/^{-}$ knockout) mice, notably reducing reactive astrogliosis and microgliosis—effects that exceeded those achieved with an antioxidant control.	[17,25]
Ibuprofen	Negatively regulates the post-synaptic NMDA glutamate receptor	Lafora disease (myoclonic seizures)	-	In PTZ-kindled rats (a chronic seizure model), ibuprofen significantly reduced seizure severity and duration, protected hippocampal neurons, and inhibited inflammation by downregulating COX-2 and NLRP3	[17,26]

				inflammasome activation, as well as lowering IL-18 levels. In a Lafora disease mouse model, ibuprofen reduced PTZ-induced seizure sensitivity by lowering seizure severity and duration, while also downregulating proinflammatory mediators.	

Supplementary Table S3. Clinical Profile of Emerging Anti-epileptic Drugs.

Names	Mechanism (s)	Clinical indications	Sponsor	Phase	Status NCT identifier	Start date End date	Final outcome(s)	References
Novel therapies								
Padsevonil	Binds to SV2 presynaptically (SV2A, SV2B, and SV2C), and to the benzodiazepine site on postsynaptic $GABA_A$ receptors	Drug-resistant focal-onset seizures	UCB Biopharma S.P.R.L.	2	Completed NCT03373383	2018-02-12 2020-01-30	The primary outcomes did not demonstrate statistical significance in any of the padsevonil dose groups compared to placebo. Padsevonil was generally well tolerated, with no new safety concerns observed.	[27,28]
Ganaxolone	Modulates $GABA_A$ receptors	Drug-resistant partial-onset seizures	Marinus Pharmaceuticals	3	Completed NCT01963208	2013-10 2016-10	No peer-reviewed publications are available. According to a company press release, ganaxolone did not meet the primary efficacy endpoint (median seizure reduction: 21.28% vs. 10.25% for placebo; $p = 0.1537$), and that the drug was generally safe and well tolerated.	[3,29–31]
CVL-865	A positive allosteric modulator of $GABA_A$ receptors	Focal-onset seizures	Cerevel Therapeutics, LLC	2	Completed NCT04244175	2020-01-27 2024-05-21	No outcomes are publicly available.	[3,28,30,31]
ACT-709478	Blocks three T-type calcium	Photosensitive epilepsy	Idorsia Pharmaceuticals Ltd.	2	Completed NCT03239691	2017-10-06 2018-04-25	No outcomes are publicly available.	[3,28,30]

	channel sub-types							
CX-8998	Blocks the Ca_v3 receptor	Absence seizures	Jazz Pharmaceuticals	2	Completed NCT03406702	2018-02-25 2019-03-29	No outcomes are publicly available.	[3,28,30]
EPX-100	Serotonergic (5-HT) agonist	Dravet Syndrome	Epygenix	2	Recruiting NCT04462770	2020-09-15 2029-05-01	The study is currently ongoing, no results have been published or presented.	[3,30]
XEN-1101	Potassium channel opener, deactivates the voltage-gated M-current	Primary generalized tonic- clonic seizures; focal-onset seizures	Xenon Pharmaceuticals Inc.	3	Recruiting NCT05667142	2023-02-14 2025-10	The study is currently ongoing, no results have been published or presented.	[3,9,28,30]
				3	Recruiting NCT05614063	2022-11-18 2026-02	The study is currently ongoing, no results have been published or presented.	
				3	Recruiting NCT05716100	2023-05-09 2025-12	The study is currently ongoing, no results have been published or presented.	
BHV-7000 (KB-3061)	KCNQ potassium channel (Kv7.2/7.3) activator	Refractory focal-onset epilepsy; Idiopathic generalized epilepsy with generalized tonic-clonic seizures	Biohaven Therapeutics Ltd.	2/3	Recruiting NCT06309966	2024-05-13 2025-09	The study is currently ongoing, no results have been published or presented.	[9,10]
				2/3	Recruiting NCT06132893	2024-03-14 2025-09	The study is currently ongoing, no results have been published or presented.	
				2/3	Recruiting NCT06425159	2024-06-20 2027-07	The study is currently ongoing, no results have been published or presented.	
NBI-921352 (XEN-901)	Sodium ($Na_v1.6$) channel inhibitor	SCN8A developmental and epileptic encephalopathy syndrome (SCN8A-DEE); focal-onset seizures	Neurocrine Biosciences	2	Active, not recruiting NCT04873869	2022-01-31 2025-12	The study is currently ongoing, no results have been published or presented.	[3,9,28]
				2	Terminated (the parent study (NCT0515990	2022-11-09 2024-03-11	No outcomes are publicly available.	

					8-completed) failed to meet its primary endpoint) NCT05493293			
Soticlestat (TAK-935, OV-935)	Cholesterol 24-hydroxylase inhibitor	Rare epilepsies; Lennox-Gastaut syndrome; Dravet syndrome	Takeda	2	Active, not recruiting NCT03635073	2018-07-19 2026-05-22	The study is currently ongoing, no results have been published or presented.	[3,9,28,32,33]
				3	Recruiting NCT05163314	2022-03-04 2026-05-22	The study is currently ongoing, no results have been published or presented.	
				3	Completed NCT04938427	2021-11-08 2024-01-25	No peer-reviewed publications are available. According to company's clinical study report, soticlestat did not significantly reduce MMD seizure frequency compared to placebo in the overall LGS population evaluated in this study. Soticlestat was generally well tolerated at doses up to 300 mg BID in participants with LGS.	
				3	Completed NCT04940624	2021-10-28 2024-04-11	Although the primary endpoint narrowly missed significance, the overall results support the clinical relevance of soticlestat for treating seizures in patients with DS aged ≥2 years, with consistent efficacy across endpoints and a favorable safety profile.	
TIL-TC150	Inhibits the GPR55 receptor	Pediatric epilepsy	The Hospital for Sick Children	1	Completed NCT02983695	2017-02-02 2021-07-20	No peer-reviewed publications are available. An open-label pilot phase of the trial reported safety, reduction in seizure counts, spike index on EEG, and improved quality of	[30,34]

							life measures in children with Dravet syndrome using a CBD/THC oil.	
E2730	GAT1 inhibitor	Photosensitive epilepsy	Eisai Inc.	2	Terminated (due to efficacy reasons) NCT03603639	2018-07-27 2019-02-14	No outcomes are publicly available.	[3,9]
LP-352 (Bexicaserin)	5-HT2C receptor superagonist	Dravet syndrome; developmental and epileptic encephalopathies	Longboard Pharmaceuticals	3	Recruiting NCT06660394	2024-10-02 2026-10	The study is currently ongoing, no results have been published or presented.	[35]
				3	Recruiting NCT06719141	2024-11-11 2026-11	The study is currently ongoing, no results have been published or presented.	
				2	Completed NCT05626634	2022-11-08 2024-12-20	No outcomes are publicly available.	
				1/2	Completed NCT05364021	2022-03-03 2023-11-20	No peer-reviewed publications are available. According to AES abstract, LP352 showed a favorable benefit-risk profile in a broad DEE population, with ≥75% reductions in Clinician-Made Severity (CMS) score observed in about one-third of participants, compared to none on placebo.	
AMT-260	AAV9 vector delivering microRNA silencing technology to degrade the GRIK2 gene and downregulate the expression of glutamate receptor subtype GLUK2	Unilateral refractory mesial temporal lobe epilepsy	UniQure Biopharma B.V.	1/2	Recruiting NCT06063850	2024-06-12 2031-12	The study is currently ongoing, no peer-reviewed publications are available. According to a company press release, early results report no serious adverse events and 92% reduction in seizure frequency.	[9,10,36]

STK-001	Antisense oligonucleotide therapy – upregulates the expression of SCN1A gene and restores levels of the Nav1.1 sodium channel	Dravet syndrome	Stoke Therapeutics, Inc	2	Enrolling by invitation NCT04740476	2021-01-20 2027-03-03	The study is currently ongoing, no peer-reviewed publications are available. According to AES abstract, STK-001–treated patients showed sustained reductions in convulsive seizure frequency and meaningful improvements in cognitive and behavioral measures.	[9,30,31,37–39]
				1/2	Completed NCT04442295	2020-06-29 2023-11-14	No peer-reviewed publications are available. According to AES abstracts, STK-001 was generally well tolerated, with a favorable benefit-risk profile observed at single and multiple doses up to 70 mg. Spectral EEG analysis demonstrated a sustained effect of STK-001, suggesting an improved cognitive function.	
RAP-219	TARP γ-8 negative allosteric modulator, selectively and potently inhibits AMPA receptors containing TARP γ-8	Refractory focal epilepsy	Rapport Therapeutics Inc.	2	Recruiting NCT06377930	2024-10-10 2025-06-01	The study is currently ongoing, no results have been published or presented.	[10]
ENX-101	α2/3/5 subtype-selective positive allosteric modulator $GABA_A$ receptor	Focal seizures	Engrail Therapeutics INC	2	Withdrawn (due to business reasons) NCT05481905	2022-09 2024-12	No outcomes are publicly available.	[31]

ETX-101	AAV vector that encodes a sodium channel subunit (Nav1.1) and restores the function of GABAergic interneurons	Dravet syndrome	Encoded Therapeutics	1/2	Recruiting NCT05419492	2024-05-14 2031-04	The study is currently ongoing, no results have been published or presented.	[10,30,31]
				1/2	Recruiting NCT06112275	2024-02-28 2030-06	The study is currently ongoing, no results have been published or presented.	
				1/2	Recruiting NCT06283212	2024-05-09 2029-12	The study is currently ongoing, no results have been published or presented.	
SPN-817 (Huperzine A)	Highly selective acetylcholinesterase (AChE) inhibitor	Refractory epilepsy	Supernus Pharmaceuticals, Inc.	2	Unknown status NCT05518578	2023-02-07 2025-01	No peer-reviewed publications are available. According to AES abstract, maintenance treatment with SPN-817 led to meaningful seizure reductions in individuals with treatment-resistant epilepsy, including focal seizures, with over half achieving a ≥50% decrease in seizure frequency. Adverse events, cholinergic in nature, were frequent during SPN-817 titration.	[9,40]
NRTX-1001	Neural cell therapy - direct transplantation of GABAergic post-mitotic inhibitory interneurons into epileptogenic brain tissue	Refractory bilateral TLE; refractory unilateral MTLE	Neurona Therapeutics	1	Recruiting NCT06422923	2024-11-14 2042-06-15	The study is currently ongoing, no peer-reviewed publications are available. According to a company press release, 92% median seizure reduction was achieved at 7-12 months post-treatment with low dose (80% achieved >75% seizure reduction), and 72% median seizure reduction was achieved at 4-9 months post-treatment with high dose (80% achieved >50% seizure reduction).	[9,31,41]
				1/2	Recruiting NCT05135091	2022-06-16 2042-02	The study is currently ongoing, no peer-reviewed publications are available. According to a company press	

							release, no serious adverse events were attributed to the cell therapy.	
JNJ-40411813 (ADX71149)	A selective positive allosteric modulator of the metabotropic Glutamate 2 receptor (mGlu2R)	Focal onset seizures	Janssen Research & Development, LLC	2	Completed NCT04836559	2021-05-18 2024-02-08	No peer-reviewed publications are available. According to a company press release, the Phase 2 trial did not meet the primary endpoint of time to reach baseline seizure count with adjunctive ADX71149, though the treatment was safe and well tolerated.	[3,28,42]
VAL-1221	Degrades cerebral polyglucosans	Lafora disease (myoclonic seizures)	Parasail, LLC	-	Available NCT05930223	2023-06-25 -	No peer-reviewed publications are available. According to AES abstract, 10 patients with Lafora disease are currently receiving VAL-1221 at 20 mg/kg every other week via intravenous infusion under the U.S. LEAP protocol and International Compassionate Use. Caregivers and physicians have reported favorable safety and encouraging case study outcomes.	[9,43]
VX-765 (Belnacasan)	Inhibits caspase-1 and IL-1β production	Refractory partial epilepsy	Vertex Pharmaceuticals Inc.	2a	Completed NCT01048255	2010-01 2010-11	No peer-reviewed publications are available. According to a company press release, VX-765 safe and tolerable, but did not demonstrate statistically significant reduction in seizures versus placebo.	[3,15,44,45]
				2b	Terminated (by sponsor) NCT01501383	2011-12 2013-08	No peer-reviewed publications are available. According to AES abstract, the study was administratively terminated and, due to the small sample size, lacked statistical power for	

							comparisons. VX-765 was generally well tolerated across all tested doses.	
Naluzotan (PRX-00023)	Dual serotonin (5-HT)$_{1A}$ receptor agonist and σ-1 receptor antagonist	Focal epilepsy	National Institute of Neurological Disorders and Stroke (NINDS)	2	Terminated NCT01281956	2011-01-07 2017-10-05	No outcomes are publicly available.	[3]
Lorcaserin (E2023)	5-HT$_{2c}$ receptor agonist	Dravet syndrome; refractory epilepsy	Eisai Inc.	-<br><br>3	Available NCT04457687<br><br>Terminated (by sponsor) NCT04572243	2020-06-30 -<br><br>2020-09-23 2024-08-15	No outcomes are publicly available.<br><br>No outcomes are publicly available.	[3]
Natalizumab	Blocks leukocyte migration across the blood-brain barrier	Focal epilepsy	Biogen	2	Completed NCT03283371	2018-03-20 2020-11-18	The phase 2 study found that natalizumab as adjunctive therapy was safe and well-tolerated but did not demonstrate a significant reduction in seizure frequency compared to placebo in people with drug-resistant focal epilepsy.	[3,30,46]
Rozanolixizumab	A humanized anti-FcRn monoclonal antibody that specifically targets the IgG-binding region of FcRn	Leucine-Rich Glioma Inactivated 1 Autoimmune Encephalitis	UCB Biopharma SRL	2	Terminated (due to enrolment challenges) NCT04875975	2021-09-27 2024-04-26	No peer-reviewed publications are available. According to a company summary, most side effects were mild or moderate, and due to the small sample size, no definitive conclusions on efficacy could be drawn.	[10,47]
Retigabine/ezogani be (XEN-496)	Voltage-gated KCNQ (Kv7.2-Kv7.5) channel opener	KCNQ DEE; partial-onset epilepsy	Xenon Pharmaceuticals Inc.<br>GlaxoSmithKline	3<br><br>4	Terminated (by sponsor) NCT04639310<br><br>Terminated (risk profile) NCT01721317	2021-03-29 2023-05-16<br><br>2012-12-19 2013-06-20	No outcomes are publicly available.<br><br>No outcomes are publicly available.	[3,28]

Anavex 2–73 (blarcamesine)	σ1 receptor agonist	Epileptic seizures associated with Rett syndrome, infantile spasms, fragile X	Anavex Life Sciences	1-3	Completed NCT04304482	2020-07-01 2023-06-30	No peer-reviewed publications are available. According to a company press release, statistically significant early behavioral improvements (RSBQ at 4 weeks) suggest potential early benefits of Anavex 2-73 in pediatric Rett patients. However, at 12 weeks, the primary endpoints fell short of statistical significance. No safety concerns were identified, and the drug was generally well tolerated.	[3,48]
Cannabidivarin (GWP-42006)	Possibly acts on GPR55 receptors or $GABA_A$ current	Focal seizures	Jazz Pharmaceuticals	2	Completed NCT02369471	2015-03 2015-11	The study did not meet its primary efficacy endpoint. GWP-42006 showed no advantage over placebo in reducing seizure frequency. GWP42006 was generally well tolerated, with mild-to-moderate adverse events consistent with known cannabinoid effects.	[30,49]
Carisbamate (YKP509)	Blocks voltage-gated sodium channels, T-type calcium channels, AMPA-receptor and NMDA-receptors mediated excitatory neurotransmission	Lennox-Gastaut syndrome; partial-onset seizures	SK Life Science, Inc.	3	Recruiting NCT05219617	2022-04-28 2026-06	The study is currently ongoing, no results have been published or presented.	[3,30,50]
				3	Completed NCT00740623	2009-01 2010-04	Adjunctive carisbamate did not show efficacy across the evaluated dose range compared to placebo in patients with partial-onset seizures. No new safety concerns were identified.	
				3	Terminated (lacked consistent efficacy data) NCT00563459	2007-11 2010-04	No outcomes are publicly available.	

Ataluren (PTC-124)	Antisense oligonucleotide therapy - selectively induces read-through of premature but abnormal stop codons, bypassing nonsense mutations	Dravet syndrome	NYU Langone Health	2	Completed NCT02758626	2016-11 2021-02-27	Ataluren showed no efficacy over placebo for treating seizures or other symptoms in children with Dravet syndrome (DS) or CDKL5 deficiency disorder (CDD) caused by nonsense variants. No treatment-related serious adverse events occurred during the double-blind phase, aligning with ataluren's favorable safety profile observed in larger studies.	[28,30,51]
Basimglurant	mGlu5 inhibitor	Epilepsy associated with tuberous sclerosis complex	Noema Pharma AG	2	Completed NCT05059327	2022-03-03 2025-04-28	No outcomes are publicly available.	[9]
Radiprodil	Negative allosteric modulator of the NR2B subunit of the NMDA receptors	Epilepsy associated with tuberous sclerosis complex, focal cortical dysplasia type II, and GRIN disorder; refractory infantile spasms	GRIN Therapeutics, Inc.;	1/2	Recruiting NCT06392009	2024-07-10 2026-07	The study is currently ongoing, no results have been published or presented.	[9,52,53]
			UCB Biopharma S.P.R.L.	1	Active, not recruiting NCT05818943	2023-03-07 2026-11	No peer-reviewed publications are available. According to AES abstract, radiprodil treatment led to a median reduction of nearly 86% in countable motor seizures during the 8-week maintenance phase, with 71% of patients achieving a ≥50% reduction. Radiprodil was generally well-tolerated.	
				2	Terminated (lack of feasibility) NCT02829827	2017-12-04 2018-10-02	Preliminary clinical testing indicated that radiprodil has a favorable safety and pharmacokinetic profile, with potential efficacy in controlling epileptic spasms. One of three treated infants achieved spasm freedom, while the other two showed clinical improvement without reaching spasm freedom.	

PRAX-628	Selectively modulates persistent sodium current and exhibits a greater activity dependence for peak sodium current, selectively targeting pathological neuronal hyperexcitability	Focal seizures, tonic-clonic seizures	Praxis Precision Medicines	2/3	Recruiting NCT06999902	2024-12-11 2025-12-08	The study is currently ongoing, no results have been published or presented.	[10]
				2	Recruiting NCT06908356	2025-01-10 2025-07	The study is currently ongoing, no results have been published or presented.	
Repurposing existing drugs								
Alprazolam	Positive allosteric modulator of $GABA_A$ receptor	Acute treatment of prolonged or repetitive seizures	UCB Biopharma SRL	3	Recruiting NCT05077904	2021-12-07 2026-04-06	No outcomes are publicly available.	[9,31,54]
				1	Completed NCT04857307	2021-04-28 2022-04-05	No peer-reviewed publications are available. According to AES abstract, alprazolam 2 mg showed rapid and highly variable absorption in adolescents with epilepsy and was well tolerated. Body weight had no meaningful impact on pharmacokinetics or safety. Modeling supports using the adult 2 mg dose in adolescents without adjustment.	
Bumetanide	Inhibits the neuronal chloride cotransporter NKCC1 that acts as a chloride importer	Phenobarbital-resistant neonatal seizures	Soul, Janet, M.D. Great Ormond Street Hospital for Children NHS Foundation Trust	1	Completed NCT00830531	2010-01 2019-01	This pilot trial demonstrated a statistically significant advantage of bumetanide over phenobarbital alone in reducing seizures, with no serious adverse events, though results were partly limited by unequal baseline seizure severity between groups.	[15,31,55,56]
				1/2	Completed NCT01434225	2011-08 2013-06	Findings indicate that adding bumetanide to phenobarbital monotherapy does not enhance seizure	

							control in newborns with hypoxic ischaemic encephalopathy and may raise the risk of hearing loss. The trial was terminated early due to serious adverse events and limited efficacy, underscoring the risks of off-label drug use in this population without prior safety evaluation in controlled trials.	
Ivermectin (EQU-001)	Positive allosteric modulator of $GABA_A$ receptor	Focal-onset seizures; Onchocerciasis-associated Epilepsy	Equilibre Biopharmaceuticals B.V.; Robert Colebunders	2	Unknown status NCT05063877	2021-08-31 2023-10-11	No peer-reviewed publications are available. According to AES abstract, PO administration of EQU-001 (60 mg) significantly ($p < 0.01$) reduced plasma levels of pro-inflammatory cytokines IL-17 and IL-1 in patients with epilepsy, compared to placebo.	[31,57,58]
				2	Terminated (insufficient funding) NCT05473442	2022-12-27 2023-07-01	No outcomes are publicly available.	
				4	Completed NCT03052998	2017-10-01 2018-08-01	Ivermectin as an add-on to anti-epileptic treatment in patients with onchocerciasis-associated epilepsy was safe and well tolerated, but did not lead to a statistically significant improvement in seizure outcomes compared to anti-epileptic drugs alone.	
N-acetylcysteine	Precursor of reduced glutathione, antioxidant, scavenger of reactive oxygen species	Refractory epilepsy	Tanta University	2	Recruiting NCT05485558	2022-09-15 2026-05-01	The study is currently ongoing, no results have been published or presented.	[15,17]
			Nationwide Children's Hospital	-	Withdrawn (no eligible subjects) NCT02054949	2013-04 2015-01	No outcomes are publicly available.	

Fingolimod	Anti-inflammatory; prevents leukocyte infiltration into the brain parenchyma; blocks sphingosine-1-phosphate receptors with broad action at the CNS	Epileptic seizures associated with Rett syndrome; Lafora disease (myoclonic seizures)	University Hospital, Basel, Switzerland	1/2	Completed NCT02061137	2013-08 2018-04	Fingolimod was safe in children with RS, but showed no clear effect on clinical, laboratory, or imaging outcomes. CSF BDNF levels correlated with clinical scores, suggesting its potential as a biomarker warrants further study.	[15,17,59]
Metformin	AMPK-dependent and AMPK-independent mechanisms; anti-inflammatory	Epileptic seizures; Lafora disease (myoclonic seizures)	Tanta University	3	Completed NCT05722951	2022-10-18 2024-06-20	No outcomes are publicly available.	[17]

Supplementary Table S4. Profile of Medicinal Plants with Anti-epileptic Potential.

Phytoconstituent(s) *Plant*	Possible mechanism(s)	Effect(s)	Type of study	References
Aconitine (alkaloid) *Aconitum*	Potentiates inhibitory response of $GABA_A$ receptor; blocks NMDA receptor; modulates sodium channels	Suppression of neuronal spike in burst; inhibition of epileptiform discharges in hippocampal slices	Preclinical (*In Vitro*)	[60–62]
Thymol (monoterpene) *Trachyspermum ammi*	Positively modulates $GABA_A$ receptor; increases chloride channel openings; blocks voltage-dependent sodium channels	The onset of convulsions was significantly delayed	Preclinical (*In Vivo*)	[62–64]
Nantenine (alkaloid) *Nandina domestica* Thunb.	Acts on calcium channels and inhibits calcium influx into the cell	Reduction of extensor to flexor ratio and mortality in maximal electric shock (MES)-induced seizure; inhibition of 90% of tonic convulsion in pentylenetetrazole (PTZ)-induced seizure	Preclinical (*In Vivo*)	[60–62]

Piperine (alkaloid) *Piper longum L.* *Piper capense L. f.* *Piper nigrum*	Antagonist of sodium channels; a positive modulatory effect on GABAergic and glycine systems; antioxidant and anti-inflammatory	Prolongation of latency to pilocarpine-induced seizures; increase in survival rate against seizures in mice model	Preclinical (*In Vivo*)	[60–62]
Bilobalide (sesquiterpene) *Ginkgo biloba L.*	Potentiates GABA synthesis	Increase in GAD activity and subsequent increase in hippocampal and cortical GABA levels	Preclinical (*In Vivo*)	[60,62]
Ginsenoside (triterpenoid saponins) *Panax ginseng Panax quinquefolius*	Inhibits NMDA receptor; regulates $GABA_A$ receptor activity	Anti-inflammatory and neuroprotective properties	Preclinical (*In Vivo*)	[62,64]
Ursolic acid stearoyl glucoside (triterpene) *Lantana camara L.*	Stimulates GABA receptor	Increase in seizure latency and decrease in seizure duration in isoniazid (INH) and tonic hind limb extension (THLE) model	Preclinical (*In Vivo*)	[60,62]
Oxypeucedanin (coumarin)	Interacts with $GABA_A$ receptor	Decrease in seizure-like behavior	Preclinical (*In Vivo*)	[62,63]
Oxypeucedanin hydrate (coumarin)	Interacts with $GABA_A$ receptor	Decrease in seizure-like behavior	Preclinical (*In Vivo*)	[62,63]
Notopterol (coumarin)	Interacts with $GABA_A$ receptor	Decrease in seizure-like behavior	Preclinical (*In Vivo*)	[62,63]
Pimpinellin (coumarin)	Interacts with $GABA_A$ receptor	Decrease in seizure-like behavior	Preclinical (*In Vivo*)	[62,63]
Hyuganin C (coumarin)	Interacts with $GABA_A$ receptor	Decrease in seizure-like behavior	Preclinical (*In Vivo*)	[62,63]
Rutin (flavonoid) *Camellia sinensis* (L.) Kuntze	A positive allosteric modulator of $GABA_A$ receptor	Increase in seizure threshold in mice; inhibition of psychomotor seizures	Preclinical (*In Vivo*)	[60,62,63]
Wogonin (flavonoid) *Scutellaria baicalensis* Georgi	Potentiates $GABA_A$ receptor	Increase in electroshock seizure threshold; decrease in electrogenic seizure severity; decrease in seizure response in PTZ-model	Preclinical (*In Vivo*)	[60]
Vitexin (flavonoid) Bambusoideae (Bamboo)	Interacts with $GABA_A$ benzodiazepine receptor complex; suppresses glutamate receptors	Increase in the latency of generalized tonic-clonic seizures and minimal clonic seizures; decrease in mortality rates in rats; decrease in seizure incidence; neuroprotective	Preclinical (*In Vivo*)	[62–64]

(+)-Erythravine (alkaloid) *Erythrina mulungu* Benth.	Modifies GABA neurotransmission	Inhibition of generalized seizures; prolongation of the latency to seizure onset	Preclinical (*In Vivo*)	[60–62]
Lobeline (alkaloid) *Lobelia nicotianaefolia*	Enhances GABA release	Protection against strychnine- and PTZ-induced seizures	Preclinical (*In Vivo*)	[62,64]
Montanine (alkaloid) *Hippeastrum vittatum* (L′H′er.) Herb.	Modulates several neurotransmitter receptor systems including $GABA_A$ receptor	Inhibition of tonic-clonic seizures	Preclinical (*In Vivo*)	[60,61]
Saikosaponin A (triterpene) *Bupleurum* plant	Blocks NMDA receptor and persistent sodium currents	Blockage of hippocampal neuron excitability; reduction in continuous epileptiform activity; inhibition of status epilepticus; blockage of NMDA-evoked current in hippocampus; reduction in severity and duration of seizures; increase in seizure latency; decrease in caspase-3 activity and apoptosis in hippocampal cells	Preclinical (*In Vivo*)	[62]
6-Gingerol (phenolic) *Z. officinale* Roscoe	Inhibits NMDA receptor via the glutamate-binding site	Improvement in EEG; decrease in locomotor activity, seizure-like behavior, and GLU/GABA ratio	Preclinical (*In Vivo*)	[63]
Magnolol (neolignan) *Magnolia officinalis*	Targets $GABA_A$, cannabinoid, and AMPA receptors	Decrease in seizure-like behavior	Preclinical (*In Vivo*)	[62,63]
Honokiol (neolignan) *Magnolia officinalis*	Targets $GABA_A$, cannabinoid, and AMPA receptors	Decrease in seizure-like behavior	Preclinical (*In Vivo*)	[62,63]
Ibogaine (alkaloid) *Tabernanthe iboga* Baill.	Blocks NMDA receptor	Reduction in THLE in mice; protection against NMDA-induced currents	Preclinical (*In Vivo*)	[60,62]
Rhynchophylline (alkaloid) *Uncaria rhynchophylla*	Blocks NMDA receptor	Reduction in NMDA- and glycine-induced maximal current responses; inhibition of NMDA receptor function in concentration dependent manner; neuroprotective	Preclinical (*In Vivo*)	[60–62]

Citronellol (monoterpenoid) *Cymbopogon winterianus* Jowitt	Activates $GABA_A$ receptor; facilitates GABA neurotransmission	Increase in clonic seizure latency; protection from tonic extension	Preclinical (*In Vivo*)	[60,64]
Dl-Tetrahydropal-matine (alkaloid) *Corydalis* yanhusuo	Reduces dopamine secretion; enhances GABAergic and cholinergic receptor functions	Decrease in release of dopamine in amygdala; reduction in behavioral excitations; may inhibit epileptic attacks	Preclinical (*In Vivo*)	[60,61]
Resveratrol	Increases expression of PGC-1α, NRF1, and Tfam; reduces S100B in CSF and serum; enhances NRF1 binding activity, COX1 level, and mtDNA amount; increases mitochondrial biogenesis; reduces caspase-3 activity; lowers ROS production; blocks TLR3	Decrease in generalized seizures, frequency of spontaneous seizures, occurrence of tonic-clonic seizures; reduction in microglia activation and neuron death	Preclinical (*In Vivo*)	[63,65]
Quercetin	Eliminates ROS; modulates antioxidant enzymes activity; increases oxidative stress marker (MDA); prevents increase in β1 and β3 subunits of $GABA_A$ receptor	Reduction in spike frequency of epileptiform activity, severity and frequency of seizures; decrease in astrocyte activation, neuroinflammation, and neuron death	Preclinical (*In Vivo*)	[63–65]
Luteolin	Increases eNOS activity; stabilizes iNOS and MMP2 activity; blocks PTZ-activated TLR4/IκBα/NF-κB pathway and TNF-α, IL-6, IL-1β synthesis; activates Akt in the hippocampus	Reduction in seizure frequency, severity, and duration; prolongation of interictal latency; suppression of neuroinflammation; neuroprotective		[64,65]
Gastrodin (phenolic) *G. elata* Blume	Enhances the expression of $GABA_A$ receptor	Reduced severity and frequency of seizure attacks in 6 cases; improvement of life quality in 7 cases; no effect in 6 cases	Clinical	[62,63,66]
Curcumin	Suppresses MAPK pathway; reduces ROS, levels of IL-6, COX-2, IL-1, NLRP3; inhibits activation of NLRP3/inflammosome; downregulates TNF-α, expression of CACNA1A and GABRD; inhibits expression of GFAP and Iba-1	Reduced the number of seizure attacks in patients with intractable epilepsy	Clinical	[65,67]

References

1. Hakami, T. Neuropharmacology of Antiseizure Drugs. *Neuropsychopharm Rep* **2021**, *41*, 336–351. https://doi.org/10.1002/npr2.12196.
2. Kanner, A.M.; Bicchi, M.M. Antiseizure Medications for Adults With Epilepsy: A Review. *JAMA* **2022**, *327*, 1269. https://doi.org/10.1001/jama.2022.3880.
3. Löscher, W.; Klein, P. The Pharmacology and Clinical Efficacy of Antiseizure Medications: From Bromide Salts to Cenobamate and Beyond. *CNS Drugs* **2021**, *35*, 935–963. https://doi.org/10.1007/s40263-021-00827-8.
4. Łukasiuk, K.; Lasoń, W. Emerging Molecular Targets for Anti-Epileptogenic and Epilepsy Modifying Drugs. *IJMS* **2023**, *24*, 2928. https://doi.org/10.3390/ijms24032928.
5. Sills, G.J.; Rogawski, M.A. Mechanisms of Action of Currently Used Antiseizure Drugs. *Neuropharmacology* **2020**, *168*, 107966. https://doi.org/10.1016/j.neuropharm.2020.107966.
6. Akyüz, E.; Köklü, B.; Ozenen, C.; Arulsamy, A.; Shaikh, Mohd.F. Elucidating the Potential Side Effects of Current Anti-Seizure Drugs for Epilepsy. *CN* **2021**, *19*, 1865–1883. https://doi.org/10.2174/1570159X19666210826125341.
7. U.S. Food and Drug Administration FDA Approves Everolimus for Tuberous Sclerosis Complex-Associated Partial-Onset Seizures Available online: https://www.fda.gov/drugs/resources-information-approved-drugs/fda-approves-everolimus-tuberous-sclerosis-complex-associated-partial-onset-seizures#:~:text=FDA%20approves%20everolimus%20for%20tuberous%20sclerosis%20complex%2Dassociated%20partial%2Donset%20seizures%20%7C%20FDA.&text=On%20April%2010%2C%202018%2C%20the%20Food%20and,with%20tuberous%20sclerosis%20complex%20(TSC)%2Dassociated%20partial%2Donset%20seizures.
8. Eliem Therapeutics *Corporate Presentation: Q1 2023 Update*; 2023;
9. Terman, S.W.; Kirkpatrick, L.; Akiyama, L.F.; Baajour, W.; Atilgan, D.; Dorotan, M.K.C.; Choi, H.W.; French, J.A. Current State of the Epilepsy Drug and Device Pipeline. *Epilepsia* **2024**, *65*, 833–845. https://doi.org/10.1111/epi.17884.
10. Bialer, M.; Johannessen, S.I.; Koepp, M.J.; Perucca, E.; Perucca, P.; Tomson, T.; White, H.S. Progress Report on New Medications for Seizures and Epilepsy: A Summary of the 17th Eilat Conference on New Antiepileptic Drugs and Devices (EILAT XVII). I. Drugs in Preclinical and Early Clinical Development. *Epilepsia* **2024**, *65*, 2831–2857. https://doi.org/10.1111/epi.18056.
11. Slassi, M.; Dove, P.; Climie, S.; O'Neill, D.; Wang, Z.; Lee, D.K.H.; De Lannoy, I.; Chen, T.; Stables, J.; Saldivia, V.; et al. FV-009 Candidate Drug: Novel and Selective P/Q-Type Ca2+ and Voltage-Gated Na+ Ion Channel Modulation for Epilepsy and Pain. *Epilepsy & Behavior* **2015**, *46*, 57. https://doi.org/10.1016/j.yebeh.2015.02.058.
12. Slassi, M.; Dove, P.; Climie, S.; O'Neill, D.; Wang, Z.; Tsirulnikov, L.; Henrianna; Lee, D.K.H.; De Lannoy, I.; Chen, T.; et al. FV-082: A Safer Orally Active Broad-Spectrum Antiepileptic Drug Candidate. *Epilepsy & Behavior* **2015**, *46*, 57–58. https://doi.org/10.1016/j.yebeh.2015.02.059.
13. Slassi, M.; Dove, P.; Climie, S.; O'Neill, D.; Wang, Z.; Tsirulnikov, L.; Henrianna; Lee, D.K.H.; De Lannoy, I.; Chen, T.; et al. FV-137 Candidate Drug: Novel and Selective Ion Channel Modulation for Epilepsy and Pain. *Epilepsy & Behavior* **2015**, *46*, 58–59. https://doi.org/10.1016/j.yebeh.2015.02.060.
14. Bar-Klein, G.; Cacheaux, L.P.; Kamintsky, L.; Prager, O.; Weissberg, I.; Schoknecht, K.; Cheng, P.; Kim, S.Y.; Wood, L.; Heinemann, U.; et al. Losartan Prevents Acquired Epilepsy via TGF-β Signaling Suppression. *Annals of Neurology* **2014**, *75*, 864–875. https://doi.org/10.1002/ana.24147.
15. Klein, P.; Friedman, A.; Hameed, M.Q.; Kaminski, R.M.; Bar-Klein, G.; Klitgaard, H.; Koepp, M.; Jozwiak, S.; Prince, D.A.; Rotenberg, A.; et al. Repurposed Molecules for Antiepileptogenesis: Missing an Opportunity to Prevent Epilepsy? *Epilepsia* **2020**, *61*, 359–386. https://doi.org/10.1111/epi.16450.

16. Leo, A.; Citraro, R.; Amodio, N.; De Sarro, C.; Gallo Cantafio, M.E.; Constanti, A.; De Sarro, G.; Russo, E. Fingolimod Exerts Only Temporary Antiepileptogenic Effects but Longer-Lasting Positive Effects on Behavior in the WAG/Rij Rat Absence Epilepsy Model. *Neurotherapeutics* **2017**, *14*, 1134–1147. https://doi.org/10.1007/s13311-017-0550-y.
17. Sanz, P.; Rubio, T.; Garcia-Gimeno, M.A. Neuroinflammation and Epilepsy: From Pathophysiology to Therapies Based on Repurposing Drugs. *IJMS* **2024**, *25*, 4161. https://doi.org/10.3390/ijms25084161.
18. Bojja, S.L.; Anand, S.; Minz, R.W.; Medhi, B. Metformin Alleviates Reactive Gliosis and Neurodegeneration, Improving Cognitive Deficit in a Rat Model of Temporal Lobe Epilepsy. *Brain Research* **2024**, *1844*, 149138. https://doi.org/10.1016/j.brainres.2024.149138.
19. Bar-Klein, G.; Klee, R.; Brandt, C.; Bankstahl, M.; Bascuñana, P.; Töllner, K.; Dalipaj, H.; Bankstahl, J.P.; Friedman, A.; Löscher, W. Isoflurane Prevents Acquired Epilepsy in Rat Models of Temporal Lobe Epilepsy. *Annals of Neurology* **2016**, *80*, 896–908. https://doi.org/10.1002/ana.24804.
20. Zubareva, O.E.; Sinyak, D.S.; Kalita, A.D.; Griflyuk, A.V.; Diespirov, G.P.; Postnikova, T.Y.; Zaitsev, A.V. Antiepileptogenic Effects of Anakinra, Lamotrigine and Their Combination in a Lithium–Pilocarpine Model of Temporal Lobe Epilepsy in Rats. *IJMS* **2023**, *24*, 15400. https://doi.org/10.3390/ijms242015400.
21. Van Vliet, E.A.; Holtman, L.; Aronica, E.; Schmitz, L.J.M.; Wadman, W.J.; Gorter, J.A. Atorvastatin Treatment during Epileptogenesis in a Rat Model for Temporal Lobe Epilepsy: Atorvastatin Treatment in Epilepsy. *Epilepsia* **2011**, *52*, 1319–1330. https://doi.org/10.1111/j.1528-1167.2011.03073.x.
22. Romariz, S.A.A.; Main, B.S.; Harvey, A.C.; Longo, B.M.; Burns, M.P. Delayed Treatment with Ceftriaxone Reverses the Enhanced Sensitivity of TBI Mice to Chemically-Induced Seizures. *PLoS ONE* **2023**, *18*, e0288363. https://doi.org/10.1371/journal.pone.0288363.
23. Wang, F.; Chen, F.; Wang, G.; Wei, S.; Fang, F.; Kang, D.; Lin, Y. Rapamycin Provides Anti-epileptogenic Effect in a Rat Model of Post-traumatic Epilepsy via Deactivation of mTOR Signaling Pathway. *Exp Ther Med* **2018**. https://doi.org/10.3892/etm.2018.6004.
24. Sandouka, S.; Singh, P.K.; Saadi, A.; Taiwo, R.O.; Sheeni, Y.; Zhang, T.; Deeb, L.; Guignet, M.; White, S.H.; Shekh-Ahmad, T. Repurposing Dimethyl Fumarate as an Antiepileptogenic and Disease-Modifying Treatment for Drug-Resistant Epilepsy. *J Transl Med* **2023**, *21*, 796. https://doi.org/10.1186/s12967-023-04695-2.
25. Mollá, B.; Heredia, M.; Sanz, P. Modulators of Neuroinflammation Have a Beneficial Effect in a Lafora Disease Mouse Model. *Mol Neurobiol* **2021**, *58*, 2508–2522. https://doi.org/10.1007/s12035-021-02285-1.
26. Liu, R.; Wu, S.; Guo, C.; Hu, Z.; Peng, J.; Guo, K.; Zhang, X.; Li, J. Ibuprofen Exerts Antiepileptic and Neuroprotective Effects in the Rat Model of Pentylenetetrazol-Induced Epilepsy via the COX-2/NLRP3/IL-18 Pathway. *Neurochem Res* **2020**, *45*, 2516–2526. https://doi.org/10.1007/s11064-020-03109-9.
27. Rademacher, M.; Toledo, M.; Van Paesschen, W.; Liow, K.K.; Milanov, I.G.; Esch, M.; Wang, N.; MacPherson, M.; Byrnes, W.J.; Minh, T.D.C.; et al. Efficacy and Safety of Adjunctive Padsevonil in Adults with Drug-resistant Focal Epilepsy: Results from Two Double-blind, Randomized, Placebo-controlled Trials. *Epilepsia Open* **2022**, *7*, 758–770. https://doi.org/10.1002/epi4.12656.
28. Hu, T.; Zhang, J.; Wang, J.; Sha, L.; Xia, Y.; Ortyl, T.C.; Tian, X.; Chen, L. Advances in Epilepsy: Mechanisms, Clinical Trials, and Drug Therapies. *J. Med. Chem.* **2023**, *66*, 4434–4467. https://doi.org/10.1021/acs.jmedchem.2c01975.
29. Marinus Pharmaceuticals Marinus Pharmaceuticals Announces Top-Line Results from Phase 3 Trial in Adult Focal Onset Seizures Available online: https://ir.marinuspharma.com/news/news-details/2016/Marinus-Pharmaceuticals-Announces-Top-Line-Results-from-Phase-3-Trial-in-Adult-Focal-Onset-Seizures/default.aspx.
30. Steriade, C.; French, J.; Devinsky, O. Epilepsy: Key Experimental Therapeutics in Early Clinical Development. *Expert Opinion on Investigational Drugs* **2020**, *29*, 373–383. https://doi.org/10.1080/13543784.2020.1743678.

31. Perucca, E.; White, H.S.; Bialer, M. New GABA-Targeting Therapies for the Treatment of Seizures and Epilepsy: II. Treatments in Clinical Development. *CNS Drugs* **2023**, *37*, 781–795. https://doi.org/10.1007/s40263-023-01025-4.
32. Takeda *Clinical Study Report: TAK-935-3002 (Redacted)*; 2024;
33. Takeda *Clinical Study Report: TAK-935-3001 (Redacted)*; 2024;
34. McCoy, B.; Wang, L.; Zak, M.; Al-Mehmadi, S.; Kabir, N.; Alhadid, K.; McDonald, K.; Zhang, G.; Sharma, R.; Whitney, R.; et al. A Prospective Open-label Trial of a CBD / THC Cannabis Oil in Dravet Syndrome. *Ann Clin Transl Neurol* **2018**, *5*, 1077–1088. https://doi.org/10.1002/acn3.621.
35. O'Brien, T.J. Additional Efficacy Analyses of the Bexicaserin PACIFIC Study in Participants with Developmental and Epileptic Encephalopathies: Responder Rates, Number Needed to Treat, and Seizure-free Days. Presented at the American Epilepsy Society, 2024.
36. uniQure uniQure Presents Clinical Case Study of First Patient Dosed with AMT-260 in Refractory Mesial Temporal Lobe Epilepsy (MTLE) Available online: https://www.globenewswire.com/news-release/2025/05/29/3090188/0/en/uniQure-Presents-Clinical-Case-Study-of-First-Patient-Dosed-with-AMT-260-in-Refractory-Mesial-Temporal-Lobe-Epilepsy-MTLE.html.
37. Sullivan, J. Patients with Dravet Syndrome in Open-Label Extension Studies of Zorevunersen (STK-001) Have Durable Reductions in Seizure Frequency and Clinically Meaningful Improvements in Cognition and Behavior. Presented at the American Epilepsy Society, 2024.
38. Cross, J.H. MONARCH and ADMIRAL Phase 1/2a Studies in U.S. and U.K. Investigating Safety and Drug Exposure of Zorevunersen (STK-001), an Antisense Oligonucleotide (ASO), in Children and Adolescents with Dravet Syndrome (DS). Presented at the American Epilepsy Society, 2023.
39. Colenbier, N. Spectral EEG Analysis Demonstrates Decreased Slow-Wave Activity in Patients with Dravet Syndrome (DS) After Treatment with STK-001, an Antisense Oligonucleotide (ASO). Presented at the American Epilepsy Society, 2024.
40. O'Brien, T.J. The RENAISSANCE Study: Interim Results of a Phase 2 Study of SPN-817 in Adults with Treatment-resistant Epilepsy. Presented at the American Epilepsy Society, 2024.
41. Neurona Therapeutics Neurona Therapeutics Presents Positive Clinical Data Update from NRTX-1001 Cell Therapy Trial in Drug-Resistant Epilepsy at American Academy of Neurology Annual Meeting Available online: https://www.neuronatherapeutics.com/news/press-releases/040825/.
42. Addex Therapeutics Addex Provides Update on ADX71149 Phase 2 Epilepsy Study Available online: https://www.addextherapeutics.com/en/investors/press-releases/addex-provides-update-adx71149-phase-2-epilepsy-study/.
43. Armstrong, D. VAL-1221: A Phase II Candidate for Lafora Disease. Presented at the American Epilepsy Society, 2023.
44. Vertex Pharmaceuticals Incorporated Vertex Announces Completion of Phase 2 Study of VX-765 in People with Epilepsy Who Did Not Respond to Previous Treatment Available online: https://investors.vrtx.com/news-releases/news-release-details/vertex-announces-completion-phase-2-study-vx-765-people-epilepsy.
45. French, J. A Randomized, Double-Blind, Placebo-Controlled Study to Evaluate the Efficacy, Safety and Tolerability of VX-765 in Patients with Treatment-Resistant Focal Seizures. Presented at the American Epilepsy Society, 2013.
46. French, J.A.; Cole, A.J.; Faught, E.; Theodore, W.H.; Vezzani, A.; Liow, K.; Halford, J.J.; Armstrong, R.; Szaflarski, J.P.; Hubbard, S.; et al. Safety and Efficacy of Natalizumab as Adjunctive Therapy for People With Drug-Resistant Epilepsy: A Phase 2 Study. *Neurology* **2021**, *97*. https://doi.org/10.1212/WNL.0000000000012766.
47. UCB Biopharma SRL *Clinical Study Results: Rozanolixizumab in Adults with LGI1 Autoimmune Encephalitis (AIE001)*; 2025;

48. Anavex Life Sciences Corp. Anavex Life Sciences Provides an Update on Rett Syndrome Program. Available online: https://www.globenewswire.com/news-release/2024/01/02/2802638/29248/en/Anavex-Life-Sciences-Provides-an-Update-on-Rett-Syndrome-Program.html.
49. Brodie, M.J.; Czapinski, P.; Pazdera, L.; Sander, J.W.; Toledo, M.; Napoles, M.; Sahebkar, F.; Schreiber, A.; on Behalf of the GWEP1330 Study Group; Nezadal, T.; et al. A Phase 2 Randomized Controlled Trial of the Efficacy and Safety of Cannabidivarin as Add-on Therapy in Participants with Inadequately Controlled Focal Seizures. *Cannabis and Cannabinoid Research* **2021**, *6*, 528–536. https://doi.org/10.1089/can.2020.0075.
50. Halford, J.J.; Ben-Menachem, E.; Kwan, P.; Ness, S.; Schmitt, J.; Eerdekens, M.; Novak, G. A Randomized, Double-blind, Placebo-controlled Study of the Efficacy, Safety, and Tolerability of Adjunctive Carisbamate Treatment in Patients with Partial-onset Seizures. *Epilepsia* **2011**, *52*, 816–825. https://doi.org/10.1111/j.1528-1167.2010.02960.x.
51. Devinsky, O.; King, L.; Bluvstein, J.; Friedman, D. Ataluren for Drug-resistant Epilepsy in Nonsense Variant-mediated Dravet Syndrome and CDKL5 Deficiency Disorder. *Ann Clin Transl Neurol* **2021**, *8*, 639–644. https://doi.org/10.1002/acn3.51306.
52. Panzara, M. Pharmacokinetics, Safety/Tolerability, and Effect on Seizure Frequency and Behavior of Individually Titrated Radiprodil Doses in Children with Grin-Related Disorder: Top Line Multicenter Study Data. Presented at the American Epilepsy Society, 2024.
53. Auvin, S.; Dozières-Puyravel, B.; Avbersek, A.; Sciberras, D.; Collier, J.; Leclercq, K.; Mares, P.; Kaminski, R.M.; Muglia, P. Radiprodil, a NR2B Negative Allosteric Modulator, from Bench to Bedside in Infantile Spasm Syndrome. *Ann Clin Transl Neurol* **2020**, *7*, 343–352. https://doi.org/10.1002/acn3.50998.
54. Klein, P. Pharmacokinetics and Tolerability of Single-Dose Staccato® Alprazolam in Adolescents with Epilepsy and Population PK Analysis to Support Dose Selection in Adolescents. Presented at the American Epilepsy Society, 2023.
55. Soul, J.S.; Bergin, A.M.; Stopp, C.; Hayes, B.; Singh, A.; Fortuno, C.R.; O'Reilly, D.; Krishnamoorthy, K.; Jensen, F.E.; Rofeberg, V.; et al. A Pilot Randomized, Controlled, Double-Blind Trial of Bumetanide to Treat Neonatal Seizures. *Annals of Neurology* **2021**, *89*, 327–340. https://doi.org/10.1002/ana.25959.
56. Pressler, R.M.; Boylan, G.B.; Marlow, N.; Blennow, M.; Chiron, C.; Cross, J.H.; De Vries, L.S.; Hallberg, B.; Hellström-Westas, L.; Jullien, V.; et al. Bumetanide for the Treatment of Seizures in Newborn Babies with Hypoxic Ischaemic Encephalopathy (NEMO): An Open-Label, Dose Finding, and Feasibility Phase 1/2 Trial. *The Lancet Neurology* **2015**, *14*, 469–477. https://doi.org/10.1016/S1474-4422(14)70303-5.
57. Zanin-Zhorov, A. Pharmacodynamic Analysis to Evaluate Pro-Inflammatory Cytokines Levels in Peripheral Blood Samples from Patients with Epilepsy After Oral Administration of EQU-001. Presented at the American Epilepsy Society, 2023.
58. Mandro, M.; Siewe Fodjo, J.N.; Mukendi, D.; Dusabimana, A.; Menon, S.; Haesendonckx, S.; Lokonda, R.; Nakato, S.; Nyisi, F.; Abhafule, G.; et al. Ivermectin as an Adjuvant to Anti-Epileptic Treatment in Persons with Onchocerciasis-Associated Epilepsy: A Randomized Proof-of-Concept Clinical Trial. *PLoS Negl Trop Dis* **2020**, *14*, e0007966. https://doi.org/10.1371/journal.pntd.0007966.
59. Naegelin, Y.; Kuhle, J.; Schädelin, S.; Datta, A.N.; Magon, S.; Amann, M.; Barro, C.; Ramelli, G.P.; Heesom, K.; Barde, Y.-A.; et al. Fingolimod in Children with Rett Syndrome: The FINGORETT Study. *Orphanet J Rare Dis* **2021**, *16*, 19. https://doi.org/10.1186/s13023-020-01655-7.
60. Kaur, J.; Famta, P.; Famta, M.; Mehta, M.; Satija, S.; Sharma, N.; Vyas, M.; Khatik, G.L.; Chellappan, D.K.; Dua, K.; et al. Potential Anti-Epileptic Phytoconstituents: An Updated Review. *Journal of Ethnopharmacology* **2021**, *268*, 113565. https://doi.org/10.1016/j.jep.2020.113565.
61. Li, S.; Lin, X.; Duan, L. Harnessing the Power of Natural Alkaloids: The Emergent Role in Epilepsy Therapy. *Front. Pharmacol.* **2024**, *15*, 1418555. https://doi.org/10.3389/fphar.2024.1418555.

62. Malaník, M.; Čulenová, M.; Sychrová, A.; Skiba, A.; Skalicka-Woźniak, K.; Šmejkal, K. Treating Epilepsy with Natural Products: Nonsense or Possibility? *Pharmaceuticals* **2023**, *16*, 1061. https://doi.org/10.3390/ph16081061.
63. He, X.; Chen, X.; Yang, Y.; Xie, Y.; Liu, Y. Medicinal Plants for Epileptic Seizures: Phytoconstituents, Pharmacology and Mechanisms Revisited. *Journal of Ethnopharmacology* **2024**, *320*, 117386. https://doi.org/10.1016/j.jep.2023.117386.
64. Roustaei, B.; Zarezadeh, S.; Ghotbi-Ravandi, A.A. A Review on Epilepsy, Current Treatments, and Potential of Medicinal Plants as an Alternative Treatment. *Neurol Sci* **2023**, *44*, 4291–4306. https://doi.org/10.1007/s10072-023-07010-5.
65. Grabarczyk, M.; Justyńska, W.; Czpakowska, J.; Smolińska, E.; Bielenin, A.; Glabinski, A.; Szpakowski, P. Role of Plant Phytochemicals: Resveratrol, Curcumin, Luteolin and Quercetin in Demyelination, Neurodegeneration, and Epilepsy. *Antioxidants* **2024**, *13*, 1364. https://doi.org/10.3390/antiox13111364.
66. Liu, Y.; Gao, J.; Peng, M.; Meng, H.; Ma, H.; Cai, P.; Xu, Y.; Zhao, Q.; Si, G. A Review on Central Nervous System Effects of Gastrodin. *Front Pharmacol* **2018**, *9*, 24. https://doi.org/10.3389/fphar.2018.00024.
67. Erfani, M.; Ashrafzadeh, F.; Rahimi, H.R.; Ebrahimi, S.A.; Kalali, K.; Beiraghi-Toosi, M.; Faraji Rad, E. Effect of Curcumin on Pediatric Intractable Epilepsy. *Iranian Journal of Child Neurology* **2022**, *16*, 35–45. https://doi.org/10.22037/ijcn.v16i3.28525.
